# Synonymous editing alters ion channel function, favoring prime editing for retinal disease correction

**DOI:** 10.7150/ijbs.132743

**Published:** 2026-04-23

**Authors:** Meha Kabra, Mariya Moosajee, Ana Navarrete, Gregory A. Newby, Piper Rawding, Ruosen Xie, Hadas Mechoulam, Antonio Rivera, Alan Hung, Smriti Tiwari, Adam J. Waxman, Kaivalya Molugu, Krishanu Saha, Shaoqin Gong, David R. Liu, Bikash R. Pattnaik

**Affiliations:** 1 Department of Pediatrics, University of Wisconsin-Madison, Madison, WI 53792, United States.; 2 McPherson Eye Research Institute, University of Wisconsin-Madison, Madison, WI 53705, United States.; 3 Development, Ageing, and Disease, University College London (UCL) Institute of Ophthalmology, London, EC1V 9EL, United Kingdom.; 4 Department of Genetics, Moorfields Eye Hospital Health Services (NHS) Foundation Trust, London EC1V 2PD, United Kingdom.; 5 Ocular Genomics and Therapeutics Laboratory, The Francis Crick Institute, London, NW1 1AT, United Kingdom.; 6 Department of Ophthalmology, Hadassah Medical Center, Jerusalem, Israel; 7 Merkin Institute of Transformative Technologies in Healthcare, Broad Institute of Harvard and MIT, Cambridge, MA 02142, United States.; 8 Howard Hughes Medical Institute, Harvard University, Cambridge, MA 02138, United States.; 9 Department of Chemistry and Chemical Biology, Harvard University, Cambridge, MA 02138, United States.; 10 Wisconsin Institute of Discovery, University of Wisconsin-Madison, Madison, WI 53715, United States.; 11 Department of Ophthalmology and Visual Sciences, University of Wisconsin-Madison, Madison, WI 53705, United States.; 12 Department of Biomedical Engineering, Wisconsin Institute of Discovery, University of Wisconsin-Madison, Madison, WI 53706, United States.; 13 Center for Human Genomics and Precision Medicine, University of Wisconsin-Madison, Madison, WI 53705, United States.

**Keywords:** LCA16 pediatric blindness, * KCNJ13* mutation, Kir7.1, ion channelopathy, CRISPR base editing, cytosine base editing, adenine base editing, prime editing, genetic disorders, synonymous variations, human iPSC-derived retinal pigmented epithelium (RPE), stable cells.

## Abstract

Point mutations in the *KCNJ13* gene cause autosomal recessive childhood blindness, Leber congenital amaurosis (LCA16), by disrupting Kir7.1 channel function. We describe the etiology of the LCA16 retinopathy phenotype in three patients from two unrelated families harboring a homozygous *KCNJ13* missense mutation (c.431T>C, p.Leu144Pro). Our *in silico* prediction and *in vitro* validation using a human iPSC-derived retinal pigmented epithelium (RPE) model created via lipid nanoparticle-mediated delivery of the adenine base editor (ABE8e) demonstrated that the L144P mutation impairs Kir7.1 channel function and confirmed that non-viral biologic delivery is clinically translatable. We used two cytosine base editors (CBEs, BE4max-WTCas9 and evoCDA-SpCas9-NG) to correct this mutation in an L144P HEK293 stable cell model, achieving high on-target editing efficiency. However, our electrophysiological measurements showed minimal functional rescue of the channel in CBE-edited cells due to bystander nucleotide editing. Editing with evoCDA introduced a bystander missense mutation (L143F), whereas BE4max primarily generated silent mutations. Extended characterization of BE4max-edited cells revealed a distorted mRNA structure, altered half-life, and reduced abundance of cognate tRNA, all associated with these silent changes. In contrast, prime editing successfully restored channel function. Prime editors targeting the L144P locus achieved approximately 20% on-target editing without introducing bystander nucleotide editing or synonymous changes. Functional assessment demonstrated a strong genotype-phenotype correlation, with restored Kir7.1 channel activity observed in 28% of edited cells (12/43). Overall, these results highlight the importance of comprehensive functional validation of genome editing outcomes and emphasize the need for rigorous preclinical studies to translate therapeutic genome editing into first-in-human trials for genetically diverse diseases.

## Introduction

Biallelic point mutations in the *KCNJ13* gene (MIM#603208) cause autosomal recessive Leber congenital amaurosis 16 (LCA16, MIM#614186) 1-4. Allelic heterogeneity in this gene has been linked to an autosomal-dominant condition called snowflake vitreoretinal degeneration (SVD, MIM#193230) 5. The LCA16 phenotype typically presents in early childhood and is diagnosed based on clinical features, such as pigmentary abnormalities in the retina, reduced or absent electroretinogram (ERG) waveforms, nystagmus, photophobia, and progressive loss of both central and peripheral vision, ultimately leading to blindness 6, 7. The *KCNJ13* gene encodes a homotetrameric inwardly rectifying potassium channel, Kir7.1, which is expressed in the apical processes of retinal pigmented epithelial (RPE) cells. Kir7.1 channels play a critical role in helping RPE cells maintain membrane potential, uphold ionic homeostasis in the subretinal space, and facilitate the phagocytosis of the photoreceptor outer segment within RPE cells 8, 9.

Currently, there is no effective treatment available for LCA16; however, several approaches are underway to restore Kir7.1 function 2. We previously reported a case of an LCA16 patient carrying the W53X variant in the *KCNJ13* gene, which adversely affected Kir7.1 channel expression and function. Furthermore, we demonstrated the restoration of channel function using translation readthrough-inducing drugs (TRIDs) and achieved successful gene augmentation and editing in a model of LCA16 patient-derived hiPSC-RPE 9. TRID-based treatments are specifically designed for nonsense mutations and involve introducing an amino acid to replace the stop codon in the gene. Depending on the near-cognate amino acid inserted, the resulting protein may or may not yield a functional Kir7.1 channel.

In contrast, gene augmentation is a broad approach to treating mutations that cause loss of function. However, the dominant-negative effect of the mutation, as seen in SVD, and the immune response associated with lentiviral- and AAV-mediated delivery raise concerns about this method and underscore the need for new treatment options. 10. Recently developed CRISPR/Cas9 genome editors offer a therapeutic opportunity 11-14. This approach involves inducing double-stranded DNA breaks, followed by donor-dependent homology-directed repair (HDR) or non-homologous end joining (NHEJ). NHEJ produces on-target and genome-wide unintended indels, limiting its therapeutic use 15-19. For multimeric channels, different editing outcomes of recessive alleles may have complex effects. If both alleles are edited differently, with one exhibiting the desired change and the other exhibiting undesired substitutions or indels, the outcome may compromise channel function.

Most inherited ocular channelopathies are caused by point mutations 4. CRISPR base editing can reverse these mutations without requiring HDR or NHEJ. The *KCNJ13* mutations causing LCA16 and SVD are single-nucleotide changes; therefore, CRISPR-based editing with ABEs (G>A or C>T) or CBEs (T>C or A>G) could likely restore channel function 1-3, 5, 20. In this study, we tested two CBEs (BE4max-WTCas9 and evoCDA-SpCas9-NG) at the L144P mutation site (c.431T>C, p.Leu144Pro). The role of L144P in LCA16 remains unconfirmed; however, proline's rigidity disrupts secondary structure 20 and may affect the channel. We clinically evaluated three LCA16 patients harboring L144P mutations to assess disease progression and predicted the mechanism of Kir7.1 dysfunction using *in silico* tools. Lacking patient-derived RPE cells, we introduced the L144P mutation into human iPSC-derived retinal pigmented epithelium (hiPSC-RPE) using ABE8e. We showed that lipid nanoparticle (LNP)-based delivery, with an already defined FDA-approved regulatory pathway 21-23, can correct L144P, as our previous attempts with silica nanoparticles at W53X 24. The L144P mutation impairs Kir7.1 channel function in hiPSC-RPE and in stable L144P HEK293 FRT cells. Although lentivirus- and silica nanoparticle-delivered adenine base editors rescue retinal and visual functions in LCA2-RPE65 and LCA16-Kir7.1 mice, we tested cytosine base editing in stable cells and noted the need for validation given the uniqueness of the L144P mutation 21, 22. Human pro-codons (CCU, CCC, CCA, and CCG) contain two Cs, making them susceptible to CBEs causing unintended amino acid substitutions, such as Phe or Ser. We evaluated two CBEs and prime editors, analyzing electrophysiological data and mRNA stability for synonymous variants. Our findings highlight the importance of preclinical models to evaluate channel function post-editing in pathogenic channelopathies or the functionality of other proteins, as silent variations could impair rescue even after correcting the targeted mutation. We also demonstrated that prime editing can precisely correct L144P, thereby restoring function.

## Methods

### Clinical evaluation of LCA16 patients harboring the L144P mutation

Molecular testing and clinical characterization were approved by Moorfields Eye Hospital and conducted in accordance with the Declaration of Helsinki. Informed consent was obtained from all participants or data was collected as part of routine care and anonymized for publication. Ophthalmic evaluation included best-corrected visual acuity, orthoptic assessment, cycloplegic refraction, slit-lamp examination of the anterior segment, fundus examination with ultra-widefield color fundus imaging using Optos, spectral-domain optical coherence tomography (SD-OCT), standard ISCEV flash visual evoked potential (fVEP) for cortical responses, and full-field electroretinogram (ffERG) to assess rod and cone function using RETeval® as part of routine clinical care.

### Molecular genetic testing to identify the mutation

Molecular genetic testing was performed on genomic DNA extracted from blood using the retinal dystrophy-targeted gene panel at the Rare & Inherited Disease Genomic Laboratory at Great Ormond Street Hospital (London, UK) and at Blueprint Genetics (Helsinki, Finland). Coding exons and flanking intronic regions of genes, including *KCNJ13* [MIM #603208], associated with genetic eye diseases, and selected deep intronic variants were screened and analyzed as previously described 23, 24. Variant classification followed the guidelines of the American College of Medical Genetics and Genomics 25. Variants were confirmed by Sanger sequencing if they aligned with the phenotype, mode of inheritance, and family history. The datasets (variants) generated in this study were submitted to ClinVar (https://www.ncbi.nlm.nih.gov/clinvar/) (SCV001335521-SCV001335530). Written informed consent was obtained from all patients before genetic testing.

### *In silico* analysis to predict the pathogenicity of the L144P mutation

*In silico* tools were used to predict the pathogenicity of the L144P mutation. These include SIFT (Sorting Intolerant From Tolerant, https://sift.bii.a-star.edu.sg/www/SIFT_seq_submit2.html)^26^, PolyPhen-2 (Polymorphism Phenotyping v2, http://genetics.bwh.harvard.edu/pph2/)^27^, PANTHER (Protein Analysis Through Evolutionary Relationship, www.pantherdb.org) 28, SNPs & GO (https://snps.biofold.org/snps-and-go/snps-and-go.html) 29, PROVEAN (Protein Variation Effect Analyzer, http://provean.jcvi.org/index.php)^30^, and PredictSNP (https://loschmidt.chemi.muni.cz/predictsnp1/) 31. SNAP-2 (screening for non-acceptable polymorphism, https://rostlab.org/services/snap2web/) 32, a neural network-based tool, was used to generate a heatmap of every possible substitution at the L144P position of Kir7.1. To assess the stability of Kir7.1 caused by the L144P mutation, the I-Mutant tool (https://folding.biofold.org/cgi-bin/i-mutant2.0.cgi) 33 was utilized. DNASTAR (Protean-3D, www.dnastar.com/) 34 and SOPMA (https://npsa-prabi.ibcp.fr/cgi-bin/npsa_automat.pl?page=/NPSA/npsa_sopma.html)^35^ were used to assess the differences in the biophysical properties of native and mutant proteins, along with providing a 3D graphical representation of their secondary structures.

### Live-cell imaging using a heterologous expression system

HEK293 cells were cultured in a 6-well plate at 1 × 106 cells/well (75% confluence) and transfected with pAAV-eGFP-L144P Kir7.1 or pAAV-eGFP-WT Kir7.1 plasmids using TransIT-LT1 (Mirusbio #MIR 2305). Live-cell imaging was performed 24 h after transfection. Transfected cells were seeded into a 35-mm imaging dish (ibidi #81156) and stained with wheat germ agglutinin-594 (WGA-594; Thermo Fisher #W11262) to label cell membranes. Additionally, Hoechst nuclear stain (Thermo Fisher #62249) and endoplasmic reticulum tracker dye (Thermo Fisher #E34250) were used to assess protein localization.

### LNP-mediated delivery of ABE8e to WT hiPSC-RPE cells to establish the L144P mutation

LNPs were formulated using microfluidics 36 with a modified lipid mixture compared with that previously reported 37. The RNA solution was prepared in 10 mM citrate buffer (pH 5.0) by mixing ABE8e-NG mRNA (TriLink) and L144P sgRNA (GenScript, GAGGCCTAGGAGCATTTGTA, PAM-TG) at a 1:1 (wt/wt) ratio. Immediately after formulation, the LNPs were purified by ultrafiltration (Amicon, UFC905096) and subsequently characterized by dynamic light scattering (DLS) using a Zetasizer Nano instrument (Malvern Panalytical). hiPSC-RPE cells (FUJIFILM, iCell Retinal Pigment Epithelial Cells, 01279, Cat no. R1101) were cultured and maintained in transwells for 4 weeks according to the manufacturer's protocol. Mature hiPSC-RPE cells were treated with LNPs (5 µg) to create the L144P mutation. Five days after treatment, genomic DNA was extracted, and the target region was amplified using specific primers, as listed in Supplementary Table 1. Unique indexes (i7 and i5) were ligated to each amplicon by PCR, and the indexed amplicons were pooled and purified using AMPure XP beads (Beckman Coulter #A63881). The resulting library was sequenced using the NovaSeq platform. Data analysis was performed using the online tools CRISPR RGEN (http://www.rgenome.net/cas-analyzer/) 38 and CRISPResso2 (https://crispresso.pinellolab.partners.org/submission) 39.

### Characterization of HEK FRT WT and L144P stable cell lines

HEK Flp-In™ 293 stable cells (Thermo Fisher Scientific, #R75007, MA, USA) contained a single Flp recombination target (FRT) site at a transcriptionally active genomic locus and expressed the ZeocinTM gene under the control of the SV40 early promoter. The FRT site in HEK293 cells ensured stable integration and expression of the targeted protein. These cells were maintained in complete medium consisting of D-MEM (high glucose), 10% fetal bovine serum (FBS), 1% Pen-Strep, 2 mM L-glutamine, and 100 µg/ml ZeocinTM for selection. Stable WT and L144P Kir7.1 HEK293 cell lines were generated following the manufacturer's instructions. Briefly, an FLP-In™ expression vector containing a green fluorescent protein (GFP)-tagged *KCNJ13* sequence (WT or L144P) was created using in-fusion cloning. The primers used for cloning are listed in Supplementary Table 2. HEK293 FRT stable cells were co-transfected with the pOG44 recombinase expression plasmid and the FLP-In™ expression vector (Supplementary Fig. 1A) containing the *KCNJ13* sequence (WT or L144P). Forty-eight hours after transfection, cells were passaged at 25% confluence and selected for stable transfectants using 400 µg/ml hygromycin B. Hygromycin B-resistant cell clones (n = 15-20) were selected, maintained in 100 µg/ml hygromycin B, and expanded for further analysis. RNA was isolated from each clone, reverse transcribed to cDNA and subjected to Sanger sequencing with specific primers (Supplementary Table 3) to confirm the *KCNJ13* sequence (Supplementary Fig. 1B). Immunocytochemistry was performed to assess protein expression and localization (Supplementary Fig. 1C).

### gRNA design and selection

To base-edit the *KCNJ13*-L144P mutation, sgRNAs were designed using Benchling (Supplementary Fig. 2A), and a specific sgRNA-2 (GCTCCCAGGCCTCATGCTAG) was selected based on the highest on-target score (Supplementary Fig. 2B). Chemically modified sgRNA was obtained from Synthego (CA, USA).

### CRISPR-base editing of the L144P mutation using C-base editors

HEK FRT stable cells expressing GFP-tagged L144P mutant proteins were subcultured for 24 h at 70% confluence prior to nucleofection. The activities of the two cytosine base-edited mRNAs (1. BE4max-WTCas9, N1me-U modification, and 2. evoCDA-SpCas9-NG, 5moPseudoU modification) were evaluated at doses of 1, 2, and 3 µg with two sgRNAs (sgRNA-2 and sgRNA-3). Based on the results, a 3 µg dose of cytosine base editor mRNAs was selected, along with sgRNA-2 (at a 3:1 molar ratio). For base editing, 1 x 10^5 cells were electroporated using the FS-100 program on the Lonza 4D Nucleofector, following the manufacturer's guidelines. After electroporation, the cells were seeded in a 6-well plate and maintained in 100 µg/mL hygromycin B for further analysis.

### On-target and off-target analysis using deep sequencing by the Illumina platform

Five days after nucleofection, both treated and untreated cells were harvested to extract RNA (Qiagen #74134). RNA was reverse-transcribed to cDNA (Thermo Fisher #4368814) and then amplified for on-target analysis using *KCNJ13* Illumina-specific primers with adapter sequences (amplicon size ~150 bp). For off-target analysis, potential sites (Supplementary Table 4) were identified using the *in silico* tool Cas-OFFinder (http://www.rgenome.net/cas-offinder/). Parameters included an NG/NGG/NAG PAM, with or without a DNA/RNA bulge (bulge size = 1), and up to 3 mismatches to the sgRNA-2 sequence. GDNA was isolated from both treated and untreated cells and amplified with specific primers to produce 150 bp amplicons. All primer sequences are provided in Supplementary Table 1. Unique indexes (i7 and i5) were ligated to each amplicon via PCR (amplicon size 250 bp), and the indexed amplicons were pooled and purified using AMPure XP beads (Beckman Coulter #A63881). The resulting library was denatured and diluted for deep sequencing on an Illumina MiSeq platform. Deep sequencing data were analyzed using the online tools CRISPR RGEN (http://www.rgenome.net/cas-analyzer/). 38 and CRISPResso2 (https://crispresso.pinellolab.partners.org/submission) 39.

### Flow cytometry to obtain single-cell edited clones

Flow cytometry was used to isolate single cells from the edited cell pool. These individual cells were cultured to generate pure clonal cell populations with distinct edited sequences. RNA was reverse-transcribed and amplified with specific primers (Supplementary Table 3). Amplicons were sequenced by Sanger sequencing with BigDye chemistry. Edited clones with WT or synonymous changes, as well as some non-synonymous changes, were further tested for protein analysis and K+ influx.

### Protein analysis by Immunocytochemistry

Immunocytochemistry was performed to assess Kir7.1 protein expression in hiPSC-RPE (WT and edited) and HEK293 stable cells (mutant L144P, WT, pooled base-edited cells, and single-cell clones). Briefly, cells were fixed in 4% paraformaldehyde in PBS at 4°C for 10 min and washed twice with chilled PBS. Cells were permeabilized in PBS (PBST) containing 0.5% Triton X-100 at room temperature (RT) for 5 min, then incubated in a blocking buffer containing 2% BSA and 0.25% Triton X-100 for 2 h at RT. Because the protein was GFP-tagged, a GFP mouse monoclonal primary antibody (Cell Signaling #2955, 1:250) was used to detect Kir7.1 protein expression. A rabbit monoclonal primary antibody against sodium-potassium ATPase (Thermo Fisher #ST0533, 1:500) was used to label the cell membranes. Primary antibody incubation was performed overnight at 4°C. Cells were washed three times for 5 min with chilled PBS to remove unbound primary antibodies. They were then incubated with secondary antibodies, Alexa Fluor-594-conjugated Donkey anti-Rabbit (Proteintech #SA00006.8, 1:500) and Alexa Fluor-488-conjugated Donkey anti-Mouse (Proteintech #SA00006.5, 1:500), at RT for 1 h in the dark. DAPI (Biotium #40043, 1:500) was used as a nuclear counterstain. Immunostained cells were imaged with a confocal microscope (Nikon C2).

### *In silico* mRNA structure prediction and half-life

The RNAsnp web server (https://rth.dk/resources/rnasnp/) was used to predict the effects of single-nucleotide variations on mRNA secondary structure. The Kir7.1 mRNA sequence and two silent variants were input into the RNAsnp server in Mode 1. Cells were seeded in a 24-well plate and treated with actinomycin D (10 µg/mL) to block transcription and estimate the mRNA half-life. Cells were collected at time points (0, 0.5, 1, 1.5, 2, 3, and 4 h) for RNA extraction. RNA was reverse transcribed into cDNA, and real-time PCR was performed using SYBR Green chemistry. Average Ct values for each sample at each time point were normalized to the respective average Ct at t = 0 to calculate the ∆Ct value (∆Ct = average Ct at each time point - average Ct at t = 0). mRNA levels at each time point were calculated using 2(-∆CT). The mRNA decay rate and half-life were determined by nonlinear regression curve fitting (one-phase decay) using GraphPad Prism 9.

### Functional analysis of edited cells using automated patch clamp

An automated patch-clamp system (QPatch II, Sophion, Denmark) was used to measure whole-cell currents in WT, L144P, and base-edited HEK293 stable cells and hiPSC-RPE cells. Cells were randomly selected and classified as edited or non-edited based on their conductance profiles during patch-clamp recordings. For the experiment, HEK293 stable cells were grown in a T75 flask for 48-72 h and then gently detached using DetachinTM. hiPSC-RPE cells cultured on transwells were dissociated with papain, as described elsewhere. 40. The dissociated cells were centrifuged at 90 × g for 1 min and resuspended in serum-free medium containing 25 mM HEPES. The cells [3 M/ml] were kept on an instrument shaker for 20 min prior to the experiment. Forty-eight cells were recorded simultaneously using single-hole disposable Q plates, each connected to an individual amplifier. A pressure protocol was used to achieve cell positioning (-70 mbar), Giga seal (-75 mbar), and whole-cell configuration (5 pulses with a -50 mbar increase between pulses, starting at -250 mbar). The current was recorded in response to voltage-clamp steps from the holding potential (-10 mV) to voltages ranging from -150 mV to +40 mV (Δ = 10 mV). More than 60% of the cells completed the experiments. Cells with compromised stability during the experiment, as judged by leakage current, were excluded from analysis. The extracellular solution contained (in mM): 135 NaCl, 5 KCl, 10 HEPES, 10 glucose, 1.8 CaCl2, and 1 MgCl2, pH adjusted to 7.4, and osmolarity 305 mOsm. The intracellular solution contained (in mM) 30 KCl, 83 K-gluconate, 10 HEPES, 5.5 EGTA, 0.5 CaCl2, 4 Mg-ATP, and 0.1 GTP, pH adjusted to 7.2, and osmolarity of 280 mOsm. For the rubidium 'Ringer's external solution, NaCl was replaced with 140 mM RbCl, which acts as an enhancer of Kir7.1. Data analysis was performed using Sophion Analyzer versions 6.6.44 and v10.0.

### tRNA sequencing

Total RNA was extracted from HEK293 FRT WT stable cells (n = 3) and quantified using a NanoDrop ND-1000 spectrophotometer. tRNAs were isolated from total RNA, demethylated, and partially hydrolyzed following the Hydro-tRNAseq method. The tRNAs were re-phosphorylated and converted into small RNA sequencing libraries using the NEBNext® Multiplex Small RNA Library Prep Set for Illumina® kit (New England Biolabs). Size selection of PCR-amplified fragments (140-155 bp, corresponding to the 19-35 nt tRNA fragment size range) was performed. The tRNA-seq libraries were quantified using an Agilent 2100 Bioanalyzer. In accordance with the manufacturer's instructions, the libraries were sequenced for 50 cycles on the Illumina NextSeq 500 system using the NextSeq 500/550 High-Output v2 kit (75 cycles). Sequencing quality was assessed using FastQC, and trimmed reads (passing Illumina quality filters) were aligned to cytoplasmic mature tRNA sequences from GtRNAdb and mitochondrial tRNA sequences from mitotRNAdb using BWA. The number of tRNA sequences uniquely mapped to one or multiple tRNA genes was calculated using “ucount” (uniquely mapped reads overlapping the tRNA) or “mrcount” (multi-map-corrected number of reads overlapping the tRNA). For tRNA alignment, the maximum number of mismatches was 2. tRNA expression profiles were calculated based on uniquely mapped reads. Differentially expressed tRNAs were identified based on count values using the R package edgeR.

### Prime editing of L144P mutation using pegRNA and PE2max mRNA

The pegRNA was designed with the online tool pegFinder (http://pegfinder.sidichenlab.org/) and further modified by adding a “UUU” tail and incorporating 2′-OMe modifications on the first three and last three nucleotides of the RNA. Phosphorothioate bonds were introduced between these nucleotides to enhance stability and prime editing efficiency. The resulting 162-bp pegRNA sequence used in this study is listed below. In the sequence, bold letters indicate a spacer sequence, italicized letters denote an RTT (reverse transcription template) sequence, lowercase letters represent a PBS (primer-binding site) sequence, and an underlined sequence is a tevopreQ1 motif. The RNA scaffold is the sequence between the spacer and RTT. The full sequence is: AAGCCTCTAGCATGAGGCCTGTTTTAGAGCTAGAAATAGCAAGTTAAAATAAGGCTAGTCCGTTATCAACTTGAAAAAGTGGCACCGAGTCGGTGCAAATGCTCCTAGGCctcatgctagagCGCGGTTCTATCTAGTTACGCGTTAAACCAACTAGAAUUU (Supplementary Figure 3). For prime editing, HEK FRT L144P stable cells were subcultured to 70% confluence the day before nucleofection. PE2max mRNA (2 or 3 µg), pegRNA (90 or 135 pmol), and nicking guide RNA (ngRNA, 60 or 90 pmol) (Supplementary Table 5) were electroporated into 1×105 cells using the FS-100 program on a Lonza 4D Nucleofector, following the manufacturer's guidelines. After electroporation, the cells were seeded in a 6-well plate and maintained in 100 µg/ml hygromycin B for further analysis. Five days post-treatment, the cells were harvested for genetic analysis by deep sequencing on an Illumina platform.

### Statistical analysis

Each experiment was performed in triplicate with appropriate controls. Statistical analysis was performed using a two-tailed Student's t-test, and significance was set at p < 0.05. Graphs were generated using Origin 9.1 and GraphPad Prism 9.

## Results

### Patients present clinical features of LCA16

**Figure 1:**
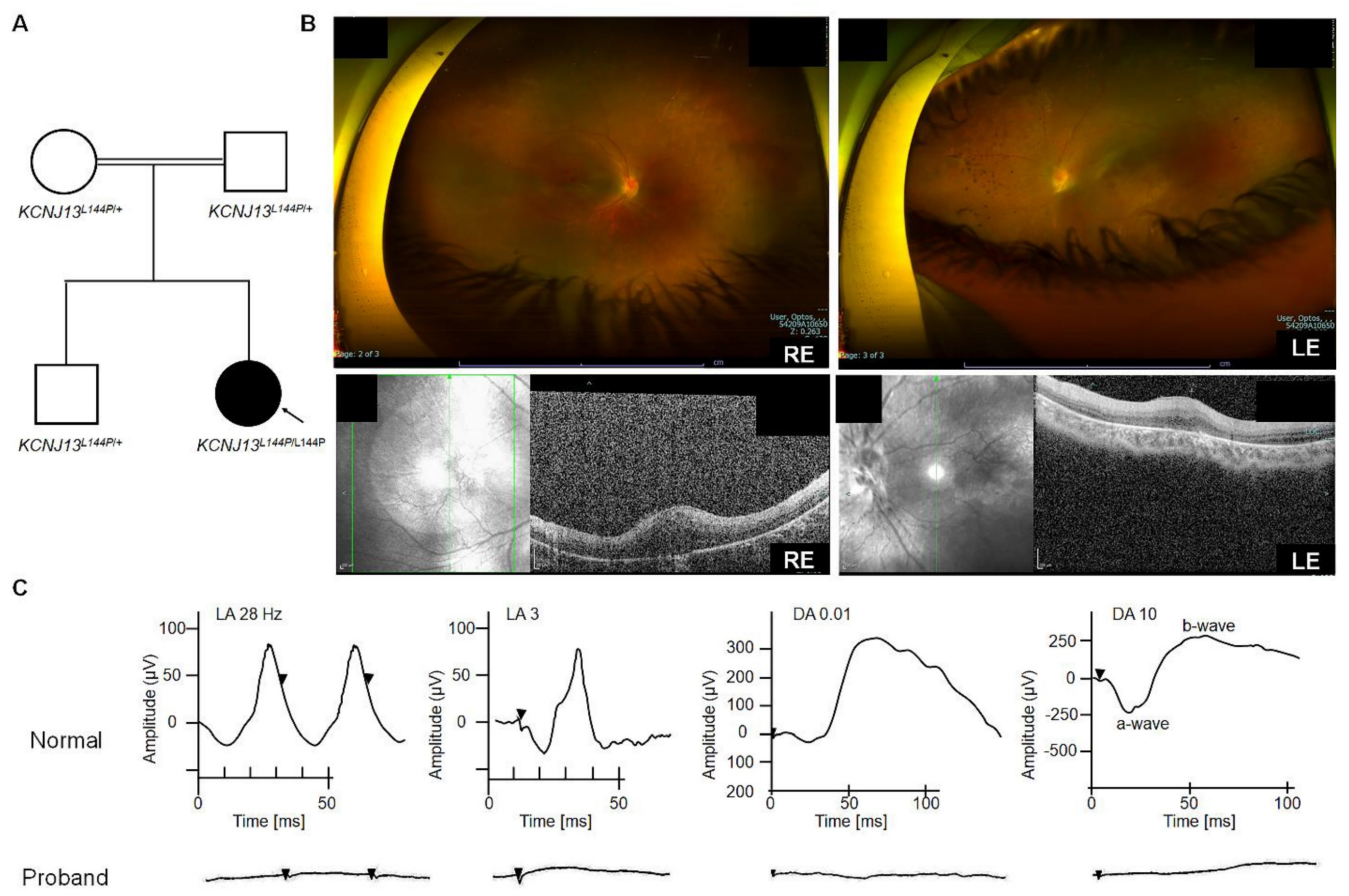
** Clinical characterization of LCA16 patients with the L144P mutation** [A] Pedigree of patient 5-1 [B] Optos and SD-OCT for Patient 5-1 [C] ffERG of patient 5-1.

One male patient (patient ID 5-1) from a consanguineous family (Arab descent) with an unremarkable prenatal and birth history was genetically diagnosed with LCA 16 (homozygous KCNJ 13 variant c. 431T>C, p. Leu 144 Pro) at age one year (2018). The patient' s older sibling and parents were heterozygous carriers and were healthy (Fig. 1A). At age one year, he exhibited nystagmus without strabismus during near and distance cover tests. Cycloplegic refraction revealed bilateral myopia with a spherical equivalent of- 6. 00 D. Eye examination also showed normal anterior segments, flat retinas with thin vascularity, peripheral pigmentary changes, and bilateral optic nerve pallor (Fig. 1B). Electrophysiological testing showed a non- recordable, flat electroretinogram (ERG) waveform on full- field ERG (ffERG) and reduced- amplitude flash visual evoked potential (fVEP) responses. The ffERG traces in both eyes confirmed widespread retinal degeneration affecting rods and cones. These findings indicated LCA or early- onset severe retinal dystrophy in the proband. At age three (2020), his best- corrected visual acuity (BCVA) was 20/500 in the right eye (RE) and 20/800 in the left eye (LE). Genetic testing of the index patient was performed with a retinal dystrophy gene panel based on a clinical exome, which uncovered a homozygous missense mutation, c. 431T>C, p. Leu 144 Pro (L144P), in the KCNJ 13 gene. The clinical parameters remained stable during the most recent visit (5 years and 3 months), with BCVA of 20/600 RE and 20/800 LE. ERG traces showed no photopic flashes or flicker responses (Fig. 1C) in either eye compared to Cone Fickler 30 Hz, with an amplitude lower than the normal limit (< 60 µV) and a normal- range time response (up to 33 ms) for a fully mature retina. Retinal function, measured as a mixed cone- rod response via ffERG (RETeval ® ISCEV protocol for flash flicker), showed no response in either eye (Fig. 1C). Even with scotopic single flashes, no b- wave was observed, possibly due to severely reduced or absent retinal activity, likely undetectable with sensor strip electrodes. Flash VEP testing involved stimulating each eye individually with a strobe light, and responses in the occipital cortex were recorded. The responses were slightly better in the right eye, with a P 100 latency of 116 ms and an amplitude of 6. 6.1 µV. For the left eye, P 100 latency was 123 ms, slightly delayed, with a symmetrical amplitude of 5. 5.5 µV. In conclusion, despite retinal vision loss, neural conduction from each eye to the occipital cortex remained intact.

**Figure 2:**
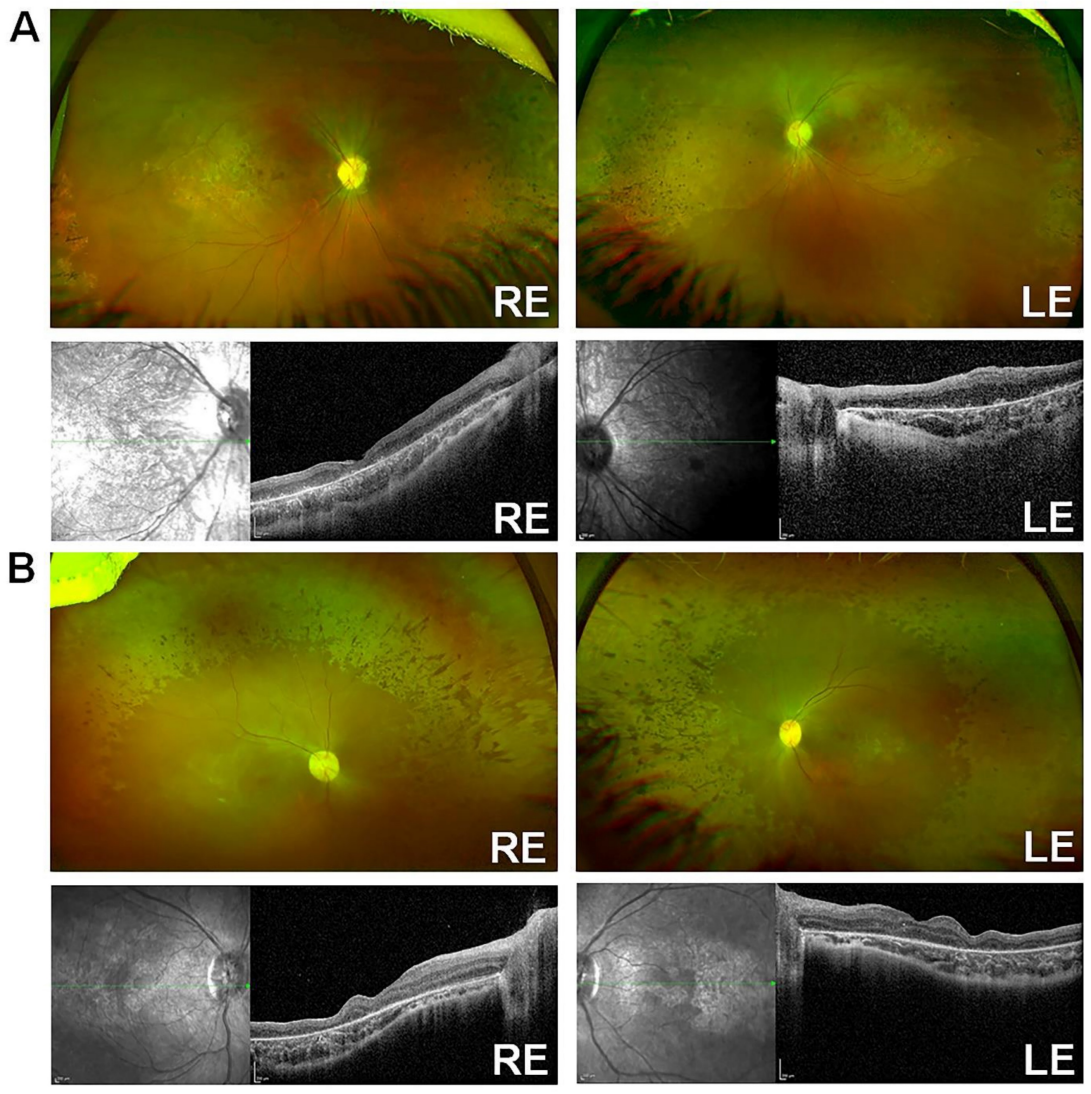
** Clinical characterization of LCA16 patients with L144P mutation** [A] Optos and SD-OCT for Patient 6-1, [B] Optos and SD-OCT for Patient 6-2.

An unrelated consanguineous family 24 (Family ID 6) of Arab descent was identified, consisting of two affected female siblings diagnosed in infancy. They are now 9 (patient ID 6-1) and 5 (patient ID 6-2) years old. Both siblings exhibited a characteristic *KCNJ13*-LCA16 phenotype, with numerous pigment spots at the RPE level, especially over the posterior pole, along with macular atrophy, optic disc pallor, and retinal vessel attenuation (Fig. 2A-B). No signs of retinovascular changes or neovascularization were observed, as previously reported 41. Patient 6-1 had rotatory nystagmus and a BCVA of 20/600 in the RE and 20/800 in the LE. Her refraction was +1.50/-2.00x170 OD and +0.50/-2.25x10 OS. Patient 6-2 exhibited horizontal nystagmus and left intermittent exotropia, with a BCVA of 20/130 in both eyes and myopic astigmatism of -5.00/-2.75x179 RE and -5.00/-2.50x175 LE. RETeval® ISCEV protocol flash- and flicker-ERG responses with sensor strip electrodes were nondetectable. SD-OCT showed extensive loss of the ellipsoid zone, retinal thinning, and disorganization in both patients (Fig. 2A-B). Both affected siblings carried the same homozygous missense variant (c.431T>C, p.Leu144Pro) in *KCNJ13*, identified through a clinical exome with a retinal dystrophy-targeted gene panel and confirmed by Sanger sequencing 24. Segregation analysis was not necessary, as the variant was homozygous in both affected sisters. No pathogenic mutations were found in any of the other genes associated with retinal disease.

### *In silico* tools predicted the L144P mutation as pathogenic

**Figure 3:**
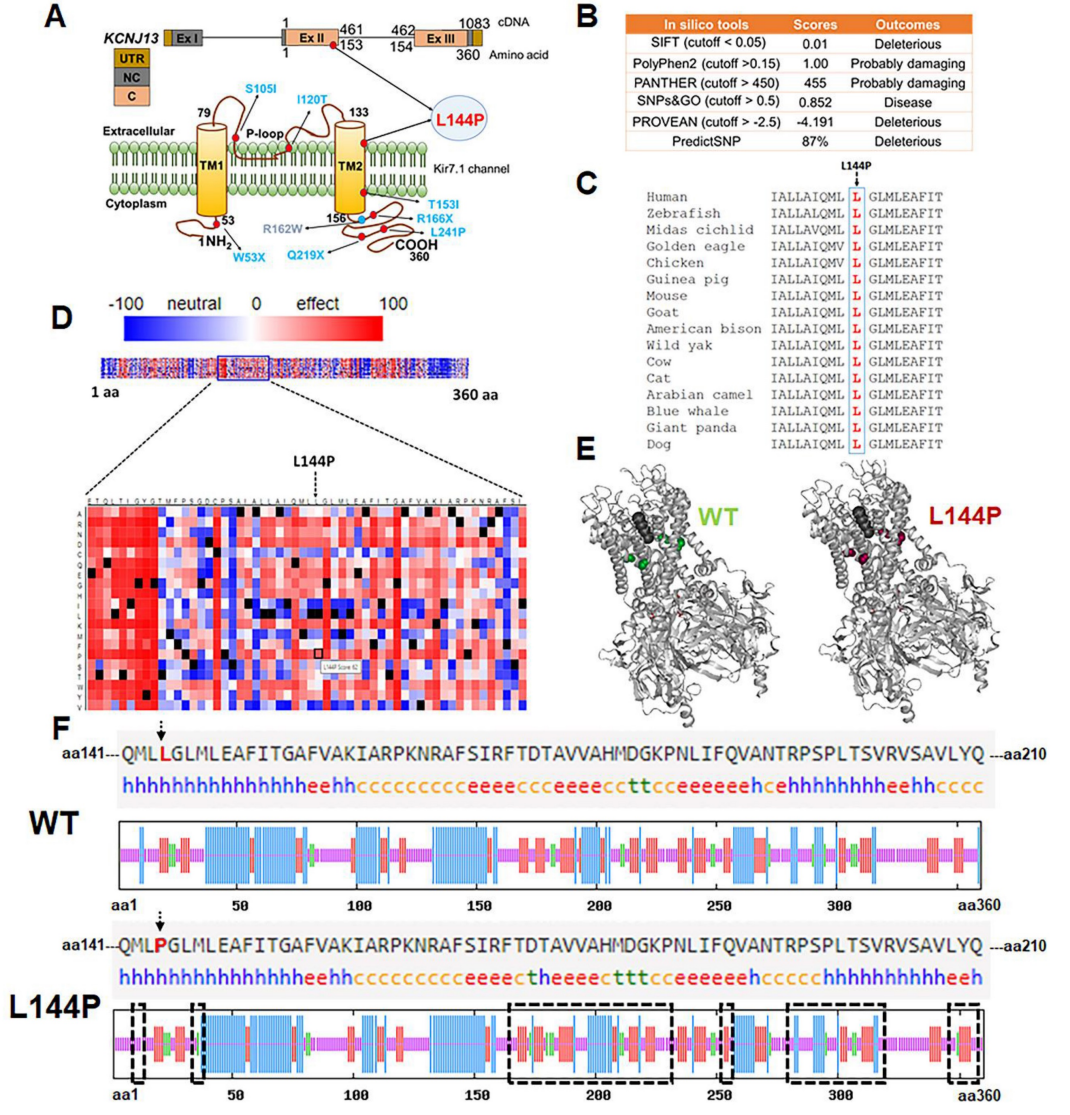
** Pathogenicity of the L144P mutation** [A] Genomic and protein location of the L144P mutation in Kir7.1. [B] *In silico* pathogenicity predictions for the L144P substitution generated using computational prediction tools. [C] Evolutionarily conserved L144 residue alignment. [D] Heatmap generated by SNAP-2 illustrating predicted functional effects of Kir7.1 L144 substitution, highlighting the pathogenic potential of L144P. [E] Secondary structure comparison of native and L144P Kir7.1 proteins predicted using DNASTAR Protean-3D. [F] Secondary structure composition predicted by SOPMA tool displays proportions of alpha-helix (blue 'h'), extended strand (red 'e'), beta-turn (green 't'), and random coil (orange 'c') in native and L144P Kir7.1. The black rectangle indicates the Kir7.1 region with a variable arrangement of secondary structures caused by the L144P substitution at the C-terminal.

Amino acid L 144 resides within the transmembrane domain- 2 (TM- 2) of Kir7.1 (Fig. 3 A), which is essential for channel gating and inward rectification. Although the change from leucine (non- polar) to proline (non- polar) does not alter the charge of the TM- 2 region, proline substitution can disrupt transmembrane α- helices and affect the protein' s structure. We used several computational tools to predict whether the L144P mutation had any structural or functional impact on the Kir7.1 channel. All seven computational algorithms (SIFT, PolyPhen- 2, PANTHER, SNPs & GO, PROVEAN, PredictSNP, and SNPA- 2) identified the L144P mutation as deleterious or disease- causing (Fig. 3B). This variant was found twice in a heterozygous state among 125, 125,455 individuals (allele frequency = 0. 0007971%) of diverse origins (gnomAD). The I- Mutant tool predicted decreased stability of Kir7.1 due to the L144P mutation, with a reliability index (RI = 4) and a free energy difference (L144P- WT = -1. 30 kcal/mol). The L 144 residue in native Kir7.1 is highly conserved across multiple species, indicating its functional relevance (Fig. 3C). A SNAP- 2- generated heatmap suggested a pathogenic signal for L144P (score = 62 and 80% accuracy), with a score >50 shown in red (Fig. 3D). The protean- 3D subroutine in DNASTAR showed minor differences in the secondary structure of Kir7.1 due to the L144P substitution, with dihedral angles changing slightly (Fig. 3E, Supplementary Table 6). Similarly, the SOPMA analysis indicated no significant differences in overall secondary- structure composition (Supplementary Table 7). Nonetheless, the L144P mutation caused notable variability (highlighted in black rectangles) at the C- terminus of Kir7.1 (Fig. 3F). The C- terminus plays a key role in membrane localization of Kir7.1 and contains putative phosphorylation sites S 287 (Protein Kinase A), T 321, and T 337 (casein kinase II) 45. Alanine substitutions at these sites did not affect Kir7.1 trafficking to the membrane. However, changes in the beta- turn and alpha- helical structures near these phosphorylation sites and in the proximal and distal C- Termini could influence cytoplasmic pore formation in the tetrameric channel and inward rectification 46-48.

### L144P substitution alters protein localization in HEK293 stable cells and hiPSC-RPE

To validate our *in silico* analysis and determine that the L144P substitution alters the localization of the Kir7.1 channel, we analyzed the cellular distribution of native and L144P Kir7.1 in HEK293 cells. As previously reported, the cell membrane showed signs of native Kir7.1 expression (Supplementary Fig. 4A) 42. However, cells transfected with the mutant eGFP-L144P-Kir7.1 plasmid exhibited fluorescence signals in the cytoplasm and other organelles, indicating that the L144P substitution impacts protein transport through the classical ER/Golgi pathway (Supplementary Fig. 4B). Staining with an ER tracker revealed that the mutant protein was mainly localized in the endoplasmic reticulum (Supplementary Fig. 4C). The absence of membrane expressions of these mutant proteins could result from a defect in ER exit.

**Figure 4:**
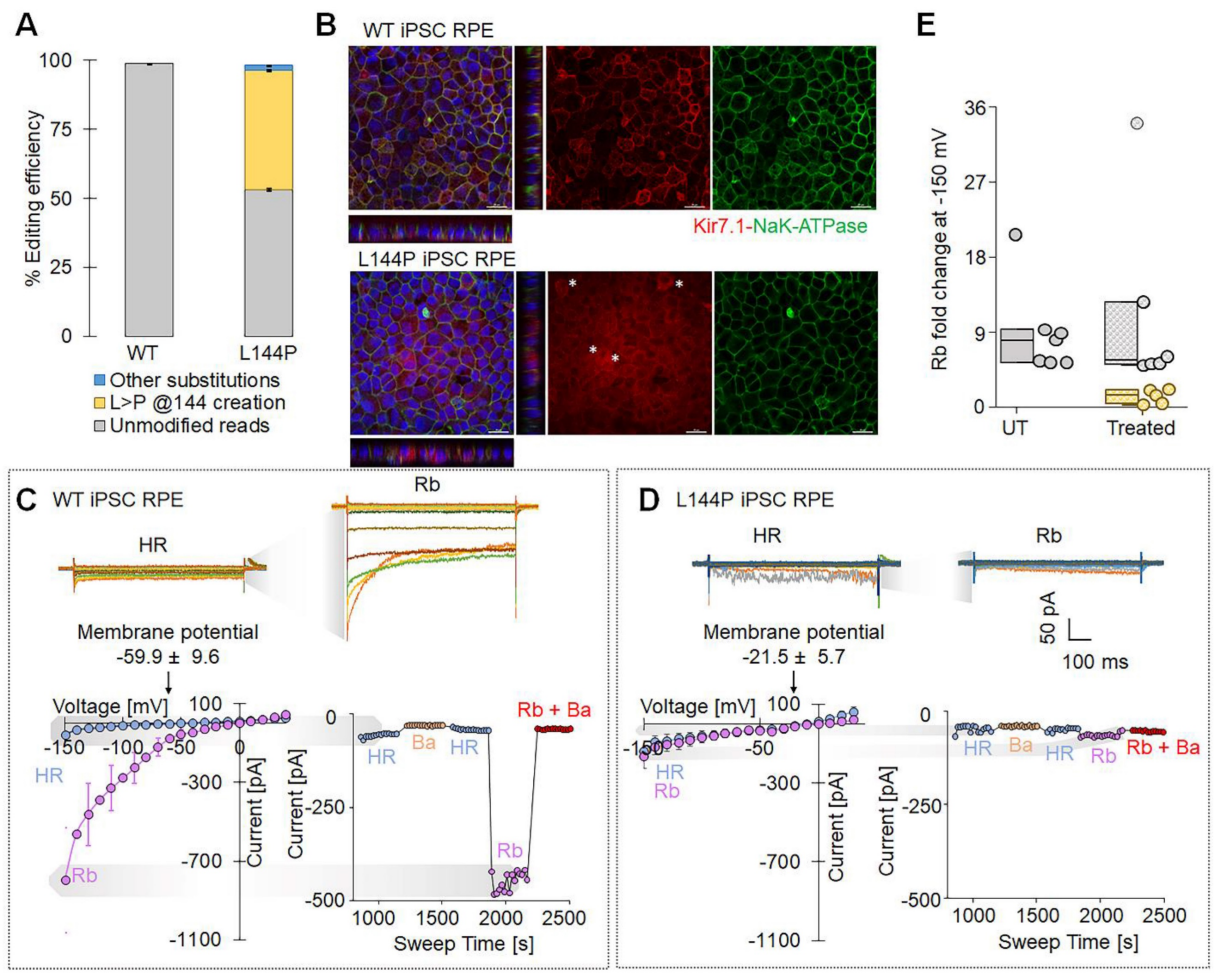
** ABE8e-mediated introduction of L144P mutation in hiPSC-RPE cells altered Kir7.1 localization and impaired channel function.** [A] Editing efficiency of ABE8e-mediated base editing used to introduce the L144P mutation into WT hiPSC-derived RPE cells (n = 3). [B] Representative immunofluorescence images of WT and L144P hiPSC-RPE monolayers stained for Kir7.1 (red), NaK-ATPase (green), and DAPI (blue). Z-stack projections illustrate the altered distribution of Kir7.1 in L144P cells. Asterisks (*) denote L144P hiPSC-RPE cells exhibiting abnormal Kir7.1 expression. Scale bar: 20 μm. [C] Upper panel: Representative whole-cell current traces showing robust inwardly rectifying K^+^ current and increased current amplitude in the presence of Rb^+^ in WT cells. Lower panel: Current-voltage (I-V) relationships recorded in HR (physiological Ringer's solution) and Rb^+^ solution demonstrating characteristic Kir7.1 -dependent inward rectification and membrane potential in WT cells. Also shown is a current-sweep plot from a representative WT cell with sequential treatments: HR (blue), 10 mM Ba^2+^ (yellow), HR wash (blue), 140 mM Rb^+^ (purple), and Rb^+^ plus Ba^2+^ (red). [D] Upper panel: Representative whole-cell current traces showing markedly reduced K^+^ current and diminished Rb^+^ response in L144P cells. Lower panel: Current-voltage (I-V) relationships demonstrating the absence of current activation in Rb^+^ solution and a compromised membrane potential in L144P cells. Also included a representative current-sweep plot illustrating sequential solution treatments. [E] Rb^+^- induced fold change in current measured in WT (n = 7), edited L144P (n = 5), and unedited cells (n = 6) within the same pool. Data are presented as mean ± SE.

To model the disease-associated L144P mutation in a human retinal context, we introduced it into WT hiPSC-RPE cells using an adenine base editor (ABE8e-NG) delivered via lipid nanoparticles (LNPs). We observed efficient base editing, with a high proportion of alleles converted to the L144P genotype (43.11 ± 0.42%) (Fig. 4A). Immunocytochemistry was performed to assess the effect of the L144P mutation on endogenous Kir7.1 expression. Compared with WT cells, a subset of edited hiPSC-RPE cells showed altered Kir7.1 localization. NaK-ATPase signal (membrane marker) remained largely intact, indicating preserved epithelial polarity and membrane integrity. Z-stack projections further demonstrated an altered distribution of Kir7.1 in edited cells, consistent with impaired trafficking, as observed in the L144P HEK293 model (Fig. 4B). We then evaluated the functional impact of the L144P mutation using high-throughput, automated whole-cell patch-clamp electrophysiology. WT hiPSC-RPE cells (n = 7) exhibited hallmark Kir7.1 electrophysiological properties, including strong inward current and a hyperpolarized resting membrane potential (-59.9 ± 9.6 mV) (Fig. 4C). In contrast, a subset of L144P-edited hiPSC-RPE cells (n = 5) showed markedly reduced inward currents and a significantly depolarized resting membrane potential (-21.5 ± 5.7 mV, p = 0.006) (Fig. 4D). Current-voltage (I-V) relationships indicated a substantial loss of inward rectification, and Ba²⁺ application caused minimal further inhibition, consistent with impaired Kir7.1 channel activity (Fig. 4D). Rb⁺ increased Kir7.1 current in WT hiPSC-RPE cells (~9-fold), whereas L144P hiPSC-RPE cells displayed a significantly attenuated response (~1.5-fold, p = 0.01), indicating impaired channel function (Fig. 4E). Notably, some cells retained WT-like Kir7.1 characteristics, likely representing unedited cells within the heterogeneous population (Rb⁺ fold change ~11-fold, n = 6, p = 0.65) (Fig. 4E). Collectively, these findings demonstrate that introducing the L144P mutation into hiPSC-RPE cells disrupts Kir7.1 membrane localization and impairs channel function.on.

### Cytosine base editor-mediated correction of the L144P mutation resulted in synonymous changes

**Figure 5:**
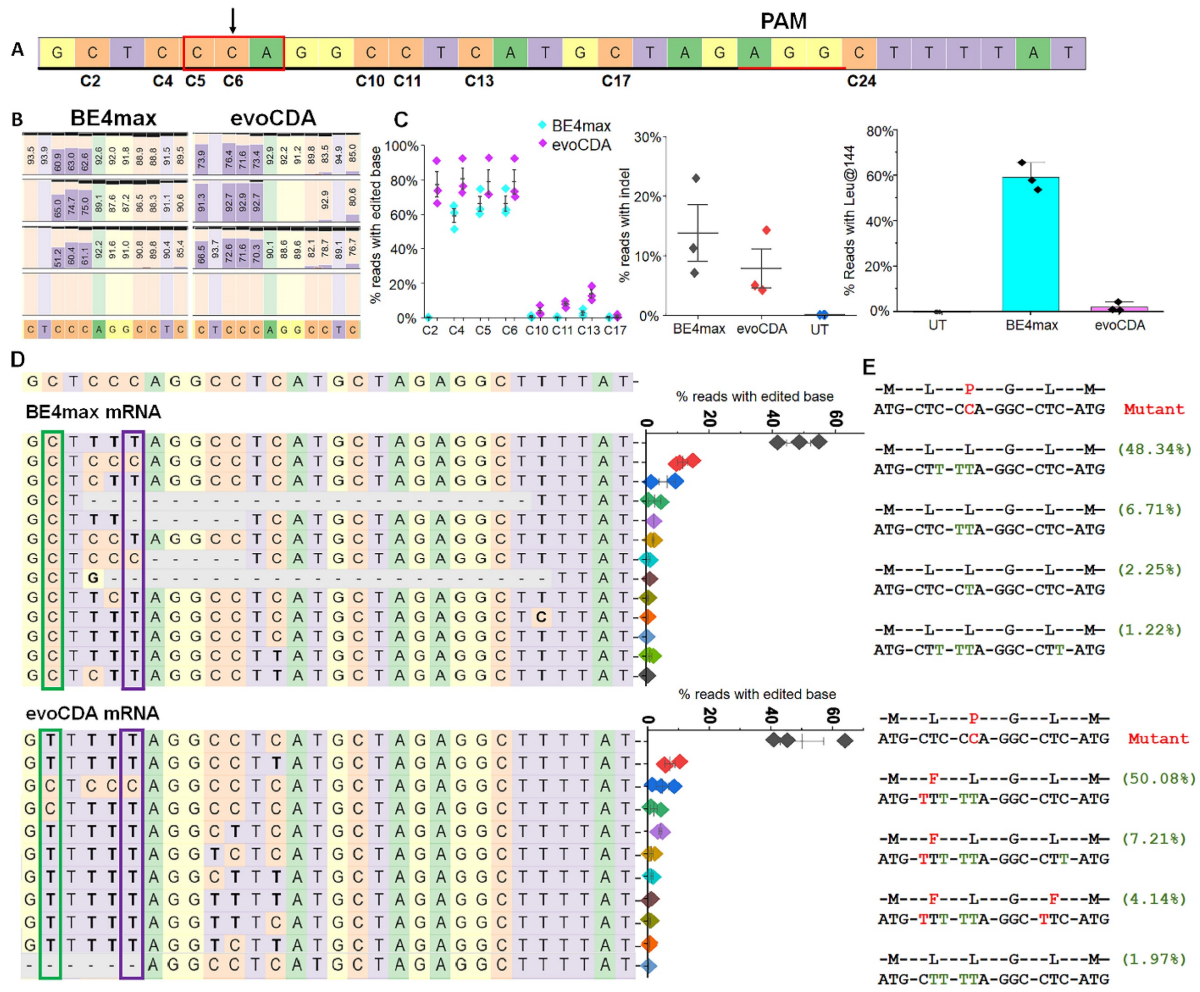
** Efficiency of CBE mRNAs (evoCDA and BE4max) in editing the L144P mutant allele and on-target analysis of CRISPR base-edited cells.** [A] Distribution of cytosines (C2-C24) surrounding the target C6 site. The sgRNA sequence is underlined in black, and the PAM is shown in red. Nucleotides are color-coded (A, green; G, yellow; C, orange; T, purple). [B] Nucleotide frequencies surrounding the sgRNA target sequence GCTCCCAGGCCTCATGCTAG in treated cells determined by sequencing. Black horizontal bars indicate the % of reads containing nucleotide deletions. [C] Editing efficiency of the target site (C6) and bystander sites (C2-C5, C10-C17) showing conversions C to T and indel frequencies following BE4max and evoCDA mRNA treatment. Data represent mean ± SEM from three independent experiments. [D] Top 10-13 reads showing nucleotide distribution around the cleavage site for sgRNA. Dashed line (-) indicates deletions and substitutions are shown in bold. BE4max mRNA-treated cells show C>T conversion at the desired site, while the C2 location remained untouched. A scatter plot displays read frequencies in the treated cells (n = 3). The green box marks C2 (aa-142) and purple box marks C6 (aa-144). The lower panel shows reads and frequencies after evoCDA mRNA treatment, in which C2 editing generates phenylalanine (F) substitutions at aa143 and aa146. [E] Amino acid conversion of the four most frequent reads, highlighting synonymous (green) and missense (red) variants generated by bystander C edits. Figures presenting pooled data are shown as mean ± SEM (n = 3).

Base editors can be used to introduce precise transition edits and reverse transition-point mutations in the genome. We tested whether this approach could improve the Kir7.1-L144P mutant phenotype by directly correcting the C>T mutation. To perform this edit, we first designed sgRNAs to target the L144P mutation in exon 2 of *KCNJ13* using two cytosine base editor (CBE) mRNAs: BE4max-WTCas9 and evoCDA-SpCas9-NG. CBE mRNAs chemically modify the targeted C to T (or G>A on the opposite strand) by engaging the deaminase domain with WT Cas9 or NG-SpCas9 and a 20-bp sgRNA. WT Cas9 primarily recognizes NGG PAMs and shows low activity toward NGA or NAG PAMs. SpCas9-NG recognizes NG PAMs but shows some activity on NA PAMs and performs best on NGNG PAMs 43, 44. The activity window of BE4max CBE mRNA ranges from positions 4 to 9, with some activity observed at positions 3 and 12 in the protospacer sequence, with the PAM counted as positions 21-23 45. The evoCDA CBE mRNA activity window spans positions 1 to 13, with peak editing observed at positions 4-6 of the protospacer 46. All three gRNAs were predicted to have off-target activity against the human genome *in silico* (Supplementary Fig. 2). In this study, we assessed the activities of sgRNA-2 and sgRNA-3 in an L144P HEK293 stable cell model. sgRNA-2 showed the highest on-target activity at the desired site (cytosine-6; C6), a key factor for efficient biallelic correction; therefore, it was selected for CRISPR base correction of L144P (Fig. 5). Conversely, sgRNA-3 did not demonstrate significant on-target efficiency (Supplementary Fig. 5).

We tested various doses of CBE mRNA (BE 4 max- WTCas 9 or evoCDA- SpCas 9- NG) and found that 3 µg was the most effective and efficient dose for editing L144P (Supplementary Fig. 6). CBE mRNA (3 µg) and chemically modified sgRNA- 2 (1 µg) were nucleofected into stable L144P mutant cells. The sgRNA- 2 guide sequence spans a region containing multiple bystander C' s, labeled C 2- C 17 within the sgRNA (Fig. 5A). First, we used genomic analysis to determine the percentage correction of the L144P missense mutation in treated cells. Reverse transcription PCR of mRNA from evoCDA- treated cells, followed by deep sequencing, showed higher editing efficiency at the desired C 6 base (78. 8% ± 7. 00) than in cells treated with BE 4 max mRNA (66. 27% ± 4. 25) (Fig. 5B). We also assessed bystander C editing in the protospacer regions. Cells edited with either editor showed bystander C editing (C 2- C 5, C 10- C 17) within the protospacer region (Fig. 5C). BE 4 max- treated cells showed a narrower editing window with only silent bystander mutations, and most reads retained Leu at position 144 (59. 0% ± 6.1. 1) (Fig. 5C). In contrast, evoCDA mRNA treatment resulted in missense bystander mutations across a broader editing window, with only ~ 2% of reads showing Leu at position 144 (Figs. 5C- 5E). Editing of C 2 (non- silent) and C 11 (non- silent) to T caused missense mutations, resulting in phenylalanine (F) at amino acid positions 143 (61. 4%) and 146 (4. 14%) in the evoCDA- treated cells. These non- synonymous C' s remained unedited in the BE 4 max- treated cells. None of the CBE- treated samples showed increased editing activity outside the protospacer. Additionally, we examined on- target indel formation by CBEs, which varied across three independent experiments. BE 4 max- treated cells showed a higher indel frequency (13. 8% ± 4. 8) than evoCDA- treated cells (8. 8.3% ± 5. 9) (Fig. 5C). Untreated cells (UT) served as a control (Supplementary Fig. 7). The efficient editing observed prompted us to proceed with functional testing of edited cells.

### Cytosine base editor-mediated correction of the L144P mutation showed protein restoration in the cell membrane

**Figure 6:**
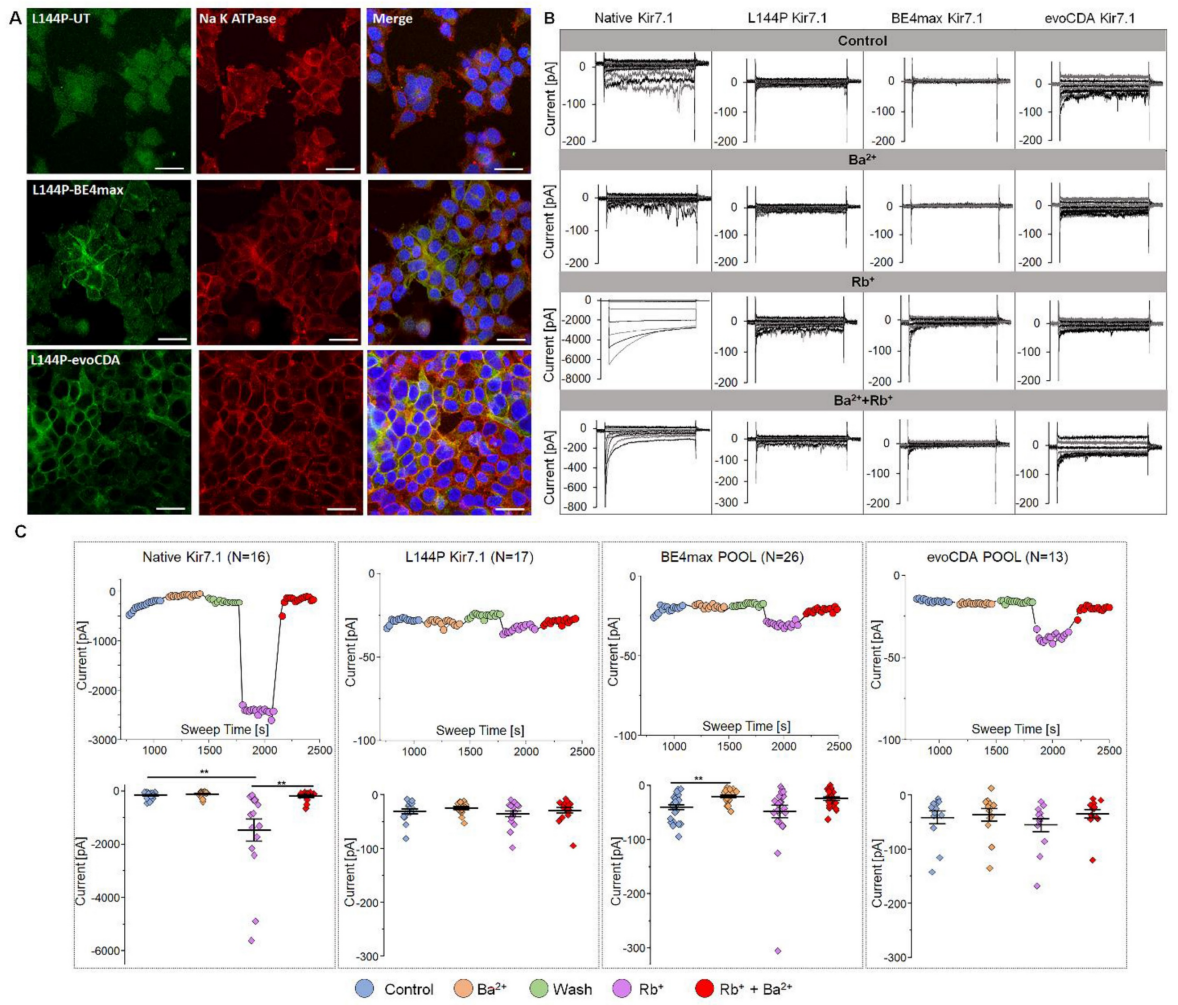
** Protein localization and K^+^ current after L144P base editing.** [A] Immunocytochemistry showing Kir7.1 protein localization in untreated and base-edited L144P cells. Scale bar: 25 μm. [B] Representative K^+^ currents recorded from WT, L144P-mutant, and base-edited cells. Currents were elicited by 550 ms voltage steps from -150 mV to +40 mV (20 mV increments), followed by a step to -10 mV (250ms). [C] Current-Sweep Time plot from a representative cell in physiological external solution (blue), 10 mM Ba^2+^ (yellow), wash with external solution (green), 140 mM Rb^+^ (purple), and Rb^+^ plus Ba^2+^ (red). The average current profile for WT, mutant, or base-edited cell pools (n = 13-26) is shown for the respective conditions. Data are presented as mean ± SEM (*p< 0.05, **p< 0.001).

Because synonymous variants are assumed not to alter protein function, we tested our CRISPR-edited cell pool to assess Kir7.1 protein levels and activity. Our immunocytochemistry assay, using a primary antibody against GFP, revealed membrane localization of Kir7.1-GFP in some cells within the BE4max-treated pool (Fig. 6A), similar to that of wild-type Kir7.1 (Supplementary Fig. 1C). Kir7.1 protein in the evoCDA-treated cell pool also showed membrane localization, despite the L143F mutation. Both edited lines showed protein restoration in some cells, which were then further examined to compare the biophysical properties of the Kir7.1 channel.

To study and test functional channel expression, whole-cell currents were measured in a 5 mM [K+] physiological extracellular solution for L144P mutant, edited, and WT cells. Cells were recorded using a 550 ms voltage pulse from -150 mV to +40 mV (20 mV increments) from a holding potential of -10 mV. The step current-voltage and current-sweep time plots for a representative cell type are shown for different treatment solutions in Figs. 6B and C. Our electrophysiological measurements revealed key features of Kir7.1 current in physiological solutions in WT cells. We observed weak inward rectification and an increase in current as WT cells hyperpolarized. The current was almost inactivated to zero when the cells were depolarized. The WT FRT stable cells produced an average Kir7.1 current of -0.16± 0.03 nA (n = 16) at -150 mV in 5 mM K+ (Fig. 6C). The inward current of the WT channel increased in the presence of external Rb+. It was inhibited by extracellular Ba2+, as expected for a normally functioning K+ channel. The Ba2+ sensitivity of the Kir7.1 channel was low, but it showed a 2-fold decrease in current (-0.11 ± 0.03 nA). On average, a ~9-fold increase in conductance (-1.46± 0.41 nA, p = 0.0063) was observed in the presence of 140 mM Rb+ external solution, while the addition of 20 mM Ba2+ to the Rb+-external solution completely inhibited Rb+ current (-0.19± 0.05 nA, p = 0.0075).

Compared with the WT channel, single-cell patch-clamp recordings of the L144P channel (n = 17) revealed a significantly lower current amplitude (-0.03 ± 0.004 nA, p = 0.001) in physiological 5 mM extracellular K+, but no further changes in current amplitude with extracellular Rb+ (-0.04 ± 0.005 nA, p = 0.52), Ba2+ (-0.02 ± 0.003 nA, p = 0.26), or Rb+ plus Ba2+ (-0.02 ± 0.004 nA, p = 0.76). Surprisingly, we did not observe restoration of K+ current in a pool of BE4max-edited cells (n = 26; -0.04 ± 0.004 nA; p = 0.15). Most cells exhibited synonymous variants, aside from the L144 correction; however, the current amplitudes were identical to those of the L144P mutant channel. These cells showed a significant block of K+ current in response to external Ba2+ (-0.01 ± 0.002 nA, p = 0.0003) but a moderate increase in Rb+ current amplitude (-0.05± 0.01 nA, p = 0.52) (Figs. 6B & C). In the pool of BE4max-edited cells, only two cells (7.7%) showed responses to K+ (-0.04 nA), Rb+ (-0.19 nA), and Ba2+ (-0.01 nA), which may be rare cells containing the WT genotype with no synonymous variations or in-frame indels. This could also result from heterotetrameric channel expression in the cell, where the stoichiometric ratio of the WT channel was equal to or greater than that with synonymous changes. The evoCDA-treated pool of cells (n = 13) also displayed a response similar to that of the mutant cells, owing to L143F and synonymous variations. There was no difference in the average current amplitudes measured at -150 mV in physiological extracellular 5 mM K+ (-0.04±0.01 nA), Ba2+ (-0.03±0.01 nA), and Rb+ (-0.05±0.01 nA). These results suggest substantial functional heterogeneity and the possibility of rare functional correction events. However, these outcomes are inconsistent and unreliable, underscoring the stochastic nature of detrimental bystander edits, which frequently introduce silent and missense mutations, resulting in a significant loss of wild-type channel function.

### L144P-targeting sgRNA exhibited fewer off-targets with BE4max mRNA

Screening for computationally predicted potential off-target (OT) sites in the human genome is crucial for assessing the safety and efficacy of CRISPR-based therapies. An *in silico* search using Cas-OFFinder 43 identified 1136 OT sites (Supplementary Fig. 8A), each with 1-3 mismatches regarding sgRNA-2, with or without a 1-nucleotide DNA/RNA bulge. Most of the identified sites had three mismatches and a single RNA bulge (n = 790), followed by three mismatches with a DNA bulge (n = 266) (Supplementary Fig. 8A and B). Our study, consistent with other published work, showed that CBEs can induce unintended genome-wide modifications. Differences in deaminase properties can affect editing profiles and efficiency 44-47. Deep sequencing of 12 putative OT sites (Supplementary Fig. 8C) showed high OT activity at OT3, OT6, and OT7 in evoCDA-treated cells. By contrast, BE4max-treated cells showed higher activity (30.75% substitution, 2.70% indel) at a single genomic location (OT3). OT3 is located in the intronic region of PDZD4 (Supplementary Table 4), more than 13 kb from the splice site. Therefore, variants at this OT site are unlikely to affect PDZD4 or Kir7.1. Reads from untreated cells showed a baseline indel rate of ~0.1%, and we set a threshold of 0.033% for our off-target assay based on base-level substitutions and indels in the reference cells. Overall, evoCDA exhibited a broad range of substitution (~0.2-51%) and indel frequencies (~0.1-4.2%) across all OT sites with detectable modifications. BE4max did not show OT activity at other sites, nor did it exceed the control treatment (Supplementary Fig. 8D).

### CBE-generated synonymous variation alters the mRNA stability and, thereby, protein synthesis

**Figure 7:**
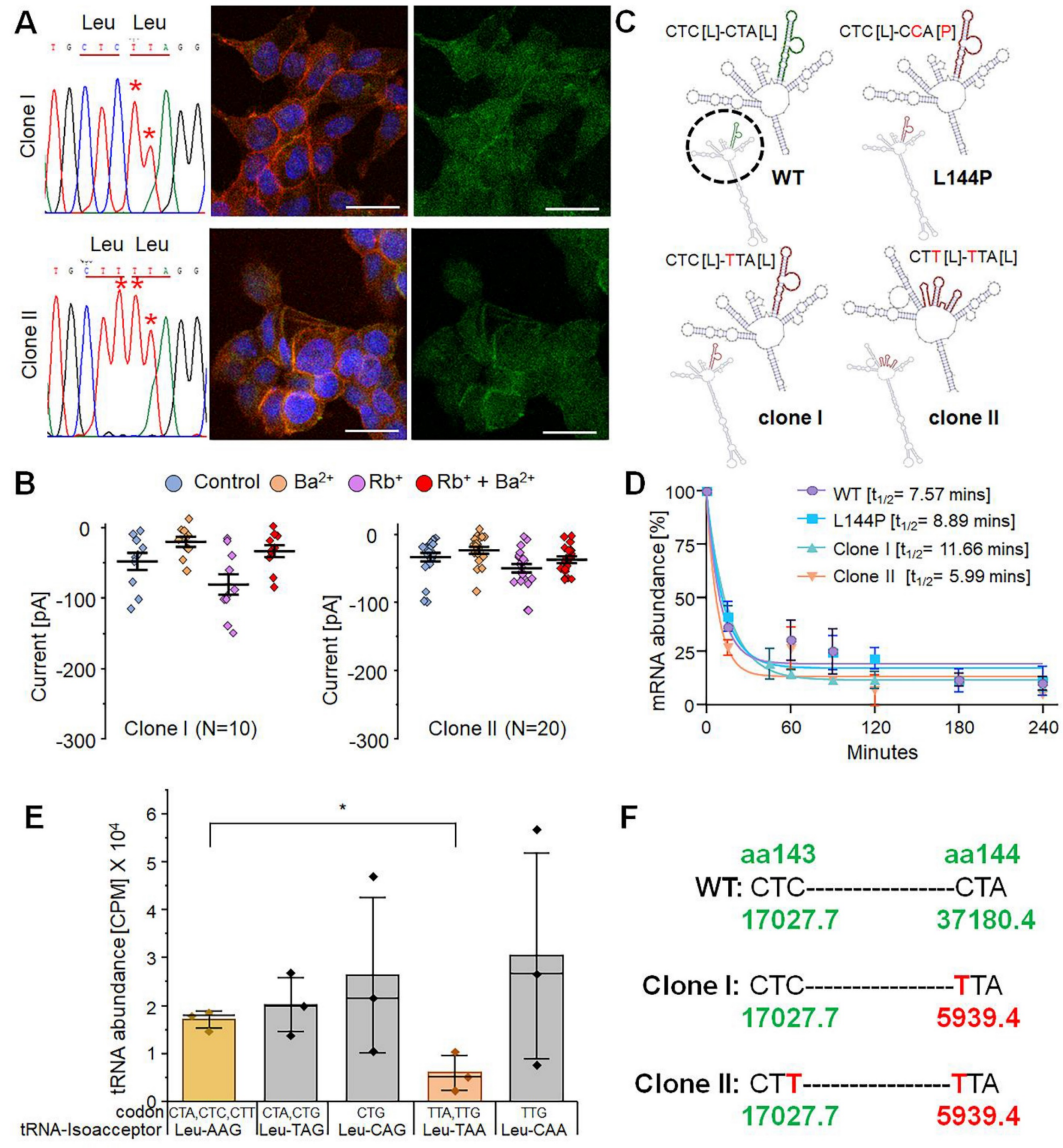
** Role of synonymous variations observed as by-products of CRISPR base editing.** [A] Sanger chromatograms of two single-cell-edited clones (clone I and clone II) and corresponding Kir7.1 expression (n = 3). Scale bar: 25 μm. [B] Representative K^+^ current profile recorded from the two single-cell clones carrying synonymous variants. [C] Predicted mRNA secondary structures for WT, L144P mutant, and the two edited clones. The enlarged region (black circle) highlights structural changes within the disruptive sequence region. [D] mRNA half-life following Actinimycin D (ActD) treatment. [E] Leu-tRNA abundance in HEK293 FRT stable cells (n = 3 biological replicates). The y-axis represents CPM (counts per million), and the x-axis shows leucine codons and their corresponding tRNA anticodons (*p < 0.05). [F] Leu-tRNA abundance (CPM) corresponding to codons at amino acid positions 143 and 144 in WT, Clone I, and Clone II cells. Pooled data are presented as mean ± SEM.

Synonymous changes are often assumed to have little effect on gene and protein function. However, multiple studies have suggested that synonymous variants can affect transcription, splicing, mRNA stability, and translation kinetics 48-50. Because we did not observe robust rescue of Kir7.1 channel function in our base-edited cells, we tested whether synonymous variants caused by bystander 'C' editing affected Kir7.1 channel activity. BE4max-edited cells were edited primarily at the target nucleotide and nearby sites, yielding only synonymous changes. Therefore, only these cells were flow-sorted to isolate single-cell clones for further analysis. Most sequence-verified clones (n = 18) were either CTC [L143]-TTA [L144] (L144bystander clone, Type I, 27.8%) or CTT [L143]-TTA [L144] (L143-L144bystander clone, Type II, 38.9%) (Fig. 7A), consistent with the pooled sequencing results. The remaining clones were either unedited or contained indels in *KCNJ13*. Clonal cells of Types I and II were expanded to study protein localization and rescue of Kir7.1 function. Immunocytochemical analysis showed that the protein was trafficked to the membrane in both clone types, although a fraction remained confined within the cytoplasm and intracellular organelles (Fig. 7A). This suggests that these silent changes might affect protein folding, disrupt oligomerization, and thus cause failure of ER quality control.

**Figure 8:**
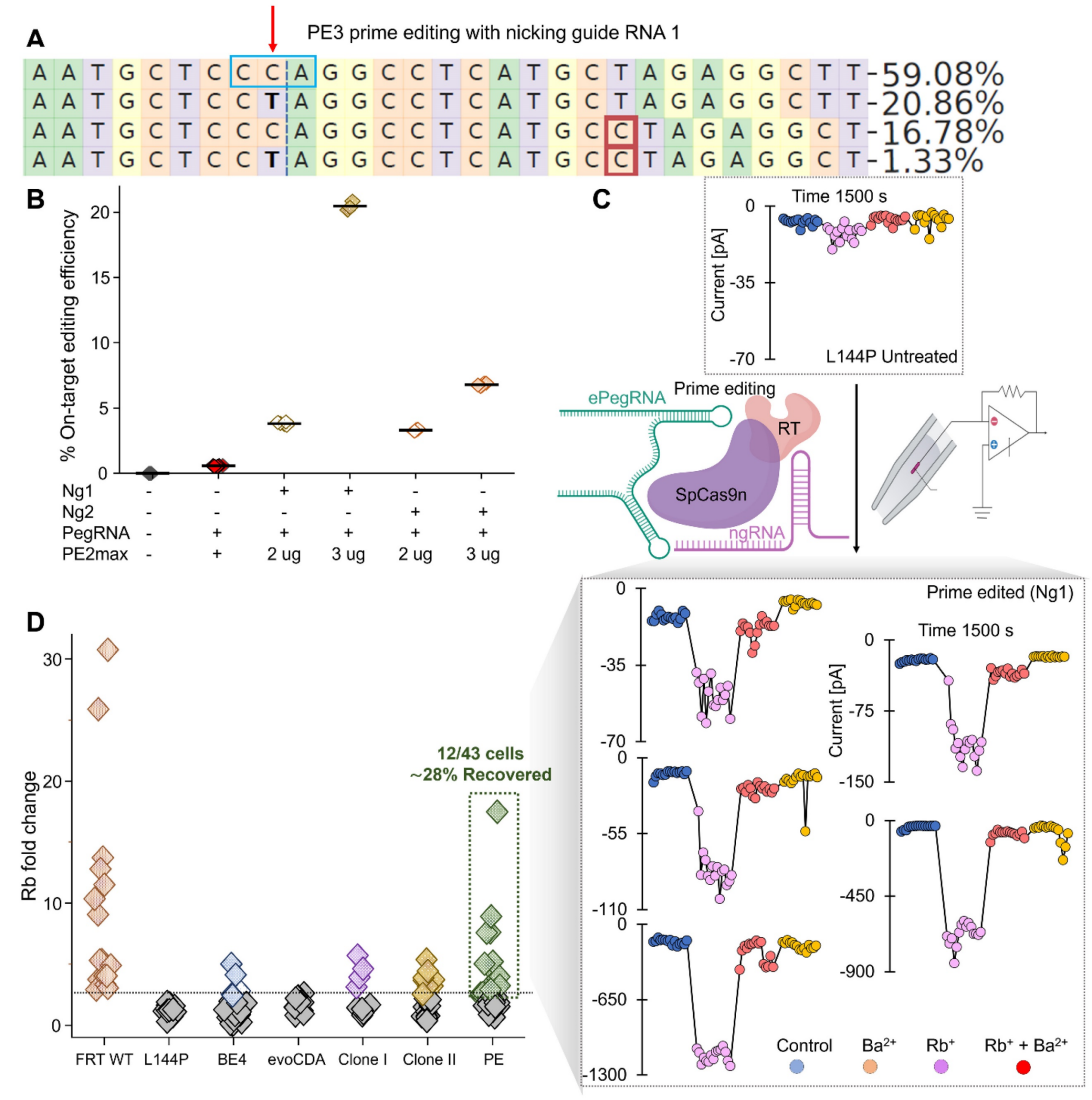
** Prime editing efficiency using the PE2 and PE3 strategies.** [A] Sequencing reads generated using the PE3 strategy with nicking guide RNA 1. The red downward arrow marks the target 'C' base, and the blue box indicates the mutant codon. The maroon box denotes an insertion, and substitutions are shown in bold. Read II shows the desired C>T conversion and its frequency. [B] On-target C>T editing efficiency at the L144P locus using the PE3 strategy with two nicking guide RNAs or without nicking gRNA (PE2 strategy) at two doses of PE2 max RNA. [C] Representative Kir7.1 current traces (current [pA] vs. sweep time [ms]) from automated electrophysiology showing L144P mutant channel responses in different external solutions and functional restoration following prime editing (PE3 with nicking guide RNA 1). Prime-edited cells showed variable K^+^ conductance under physiological conditions, increased current with Rb^+^, and reduced current with Ba^2+^ as a blocker. [D] Rb^+^ fold change across cell types: WT (n = 16), L144P (n = 17), BE4-CBE edited (n = 26), evoCDA-CBE edited (n = 13), two clones (Clone I; n = 10, Clone II; n = 20), and prime-edited cells with nicking guide gRNA 1; n = 43).

In patch-clamp experiments on edited cell clones (random selection), types I and II showed an unexpected mixed response: both edited and non-edited cells responded, whereas only edited cells were expected to respond. Approximately 35-40% of type I (n = 4/10) and type II (n = 7/20) cells displayed an approximately fourfold increase in Rb+ response (Fig. 8D), similar to that of native Kir7.1. The remaining cells from both clones showed no increase in Rb+ response. However, the physiological K+ conductance in the edited type I (-0.02 ± 0.007 nA) and type II (-0.02 ± 0.003 nA) cells was very low, unlike that of native Kir7.1. This might be due to lower overall GFP_Kir7.1 expression in the mutant L144P line compared to the WT line (Fig. 7B).

Since we did not observe robust channel function in all edited clones, we assessed whether these synonymous variants affected mRNA structure or stability. We used the *in silico* tool RNAsnp, which predicts SNP-induced changes in mRNA secondary structure using global folding 51. The Kir7.1 mRNA sequence and the two silent variants were submitted to the RNAsnp server. The tool predicted that base-pairing probabilities in the WT, L144P, and type I clones were similar, and that the mutation (CTA>CCA, L144P) or the C>T silent change (CTA>TTA, L144L) did not influence mRNA structure. However, other synonymous variants in type II clones (CTC>CTT, L143L) disrupted the mRNA structure in the region overlapping with the C>T variants (L143 and L144) (Fig. 7C). These findings suggest that structural disruption of mRNA may affect its stability and the translation of all transcripts simultaneously. To determine whether mRNA stability was codon-dependent, we treated the cells with Actinomycin D to block new mRNA transcription. We measured the decay of the existing mRNA through a time-course assay [0-4 hours] to assess mRNA abundance. This observation points to a mechanism affecting translation kinetics, co-translational protein folding, or the stability of the encoded protein, despite increased transcript stability (Fig. 7D), indicating that the functional defect cannot be explained by accelerated RNA degradation.

Another factor that could alter translation kinetics is the abundance of tRNAs for specific codons. Therefore, we examined whether there were differences in tRNA abundance for Leu codons (n = 6, CTC, CTA, CTG, TTA, TTG, and CTT) in the cells, as codon biases and differences in the availability of cognate tRNAs can affect the translation rate either directly or indirectly by slowing ribosomal progression and consequently impacting the protein co-folding mechanism. Our tRNA sequencing from HEK293 FRT stable cells (n = 3) using an Illumina NextSeq platform revealed a comparatively lower availability (counts per million, CPM) of cognate tRNA for 'Leu' (TTA), which was the codon introduced through bystander 'C' edits (Fig. 7E & 7F). A detailed mechanistic dissection of the translational dynamics underlying these effects, such as direct measurements of ribosome pausing or co-translational folding, would be required to establish a causal relationship between tRNA availability, translational pausing, and potential co-translational misfolding, and would represent an important direction for future investigation.

### Prime editing rescues Kir7.1 channel function by precisely correcting the L144P mutation

Because bystander editing can cause channel dysfunction, we explored prime editing strategies that promise to reduce bystander editing 51. Prime editors are highly efficient at correcting not only single-base mutations but also frameshift mutations (insertions, deletions, and splice-site mutations) without causing bystander nucleotide editing 52-55. We tested the PE2 (no-nicking guide RNA) and PE3 (nicking guide RNA) strategies to correct the L144P mutation in HEK FRT stable cells. We designed two nicking guide RNAs (ngRNAs) (Supplementary Fig. 3) and tested them with two doses of PE2 Max Prime Editor mRNA (2 and 3 µg). As expected, deep sequencing analysis showed higher editing efficiency with the 3 µg dose and ngRNAs (PE3 strategy) than without an ngRNA (PE2; 0.6% ± 0.01). The PE3 strategy with ngRNA 2 achieved 3.3% ± 0.03 editing efficiency at a 2 µg dose, which doubled to 6.8% ± 0.05 with a 3 µg dose. Very few sequencing reads had bystander nucleotide substitutions in the PE3-ngRNA 2 strategy (<0.3%) compared to the PE2 strategy (3%) (Supplementary Fig. 9).

Interestingly, ngRNA 1 achieved a 5-fold increase in editing efficiency (20.5% ± 0.2) with 3 µg PE2max mRNA compared with a 2 µg dose (3.8% ± 0.05) (Fig. 8A & B). Notably, among the 20% of sequencing reads with precise editing, there was no bystander nucleotide editing (Fig. 8A). A subset of reads (18.21% ± 0.05%) from cells treated with ngRNA 1 and 3 µg PE2max mRNA also displayed a single-nucleotide insertion, which occurred in both unedited (16.96% ± 0.1%) and edited reads (1.25% ± 0.04%) (Fig. 8A).

To evaluate Kir7.1 channel function, we conducted high-throughput electrophysiology on a pooled set of PE3-edited cells treated with ngRNA 1. The automated patch-clamp system randomly selected cells and labeled them “edited” based on conductance profiles observed during analysis. The current [pA] versus sweep time [ms] traces (Fig. 8C) confirmed a functional response in a subset of edited cells, with variable current amplitudes across treatment solutions. Although individual edited cells exhibited variability in K+ conductance under physiological conditions, the edited cells, on average, showed a sixfold increase in Rb+ current, confirming the restoration of Kir7.1 channel function (Fig. 8D). This variability reflects differences among cells, including variation in cell size, membrane capacitance, and the number of channels expressed at a given time. A strong genotype-phenotype correlation was evident, with 28% of edited cells (n = 12/43) displaying functional channel activity.

## Discussion

A disease is considered trial-ready when its natural history and clinically meaningful endpoints are well defined. In our study, we reported three patients with L144P-LCA16 from two unrelated Arab families who exhibited classical LCA16 features, including congenital nystagmus, early-onset vision loss, pigmentary retinopathy with degeneration of the ellipsoid zone, retinal vessel attenuation, and profoundly reduced ERGs, consistent with prior reports of the *KCNJ13*-associated LCA16 phenotype 1-3. We did not observe signs of retinal neovascularization or fibrovascular changes, as seen in patients with the p.Thr153Ile mutation, although the siblings are relatively young 41. The outcome will depend on the integrity of the RPE and the presence of clearly measurable structural and functional features.

The L144P mutation occurs in exon 2 of *KCNJ13*, which encodes the TM-2 domain of the Kir7.1 channel, a region critical for channel stability and membrane localization. Our *in silico* analyses predicted that this mutation destabilizes the protein, particularly the C-terminal region involved in trafficking. Consistent with these predictions, our heterologous expression system showed disrupted membrane localization and intracellular accumulation of mutant Kir7.1, suggesting defective folding, impaired tetrameric assembly, or altered post-translational processing. To validate these findings in a physiologically relevant system, we used LNP-mediated delivery of ABE8e to introduce the L144P mutation in hiPSC-RPE as a non-viral method with potential clinical benefit. Electrophysiological tests on L144P hiPSC-RPE and HEK293 stable cells confirmed that the L144P mutation produces a non-functional channel with impaired inward rectification. Importantly, unlike viral vectors, non-viral LNPs enable transient expression of the editing machinery, reducing long-term genotoxic risk, insertional mutagenesis, and sustained immunogenicity.

Validating genome editing for channelopathies goes beyond achieving target modification; it is about precise molecular correction, durability, functional restoration, and physiological signaling without introducing off-target alterations. The central therapeutic question our study addressed was whether genome editing could effectively restore Kir7.1 channel function in LCA16 caused by the *KCNJ13*-L144P mutation, and which editing strategy is more suitable for clinical translation. Prime editing is not preferred over base editing due to several well-documented limitations, including the need for extensive optimization, strong dependence on cellular context, generally lower editing efficiencies, and persistent challenges with efficient *in vivo* delivery 56-58. To date, only a single prime editing-based approach has advanced to a clinical trial (NCT06559176), which relies on transplanting *ex vivo* prime-edited autologous cells rather than directly correcting the target gene *in vivo* 59. Given these considerations, our study first evaluated CBEs that offer higher editing efficiency for correcting point mutations. However, restoring function to multimeric membrane proteins such as Kir7.1 presents unique challenges. Proline codons (CCU, CCC, CCA, CCG), which have two adjacent Cs, are difficult to correct without bystander edits, which may produce either silent (Leu) or missense (Ser or Phe) amino acids. Similar challenges have been reported for other disease-associated loci with clustered cytosines, such as APOE4 (Alzheimer's disease) and HBB (β-thalassemia) 60. When CBEs produced synonymous changes (Leu, silent amino acid) at the L144P locus that failed to restore Kir7.1 channel function, we explored prime editing to achieve precise correction without bystander synonymous nucleotide changes that compromise protein function.

Using two CBEs, BE4max-WTCas9 and evoCDA-SpCas9-NG, we achieved high on-target editing efficiencies (60-80%) at the L144P locus in stable cells. However, functional rescue was limited by frequent bystander edits, including a missense L143F (CTC>TTT, 61% evoCDA cells) and a synonymous L144L variant (CCA>TTA, 59% BE4max cells), both of which impaired channel function. Precise WT correction (CCA>CTA) occurred at a very low frequency (~3%) with BE4max, which also exhibited higher indel rates (⁓14%) than evoCDA, likely due to uracil excision during base excision repair. Although L144P is recessively inherited, some mutations in *KCNJ13*, such as R162W and L114P, have been reported to exert dominant-negative effects 61. Therefore, mutations or indels generated as by-products of genome editing, particularly those causing frameshifts or truncation, could lead to dominant-negative effects or haploinsufficiency. Premature termination events are often subject to quality-control mechanisms, such as nonsense-mediated mRNA decay (NMD) or protein degradation pathways, which may limit their accumulation and potential interference with properly folded channel subunits. Nevertheless, minimizing indel formation is an important safety consideration in therapeutic genome editing. Leu codon redundancy (CTA or TTA) theoretically permits restoration of the wild-type (WT) amino acid. However, these alternative outcomes were functionally detrimental. Moreover, the lack of rescue cannot be attributed to monoallelic editing, as L144P does not exert a dominant-negative effect and heterozygous individuals are asymptomatic. Off-target analysis of 12 predicted sites revealed that BE4max had greater specificity than evoCDA, with negligible activity at all other off-target sites except at a single intronic locus in PDZD4. This gene is not expressed in the RPE or other retinal layers, indicating minimal functional risk.

Together, our findings indicate that the absence of functional recovery in BE4max-edited cells is primarily driven by synonymous bystander nucleotide variants rather than by insufficient editing or off-target effects. Our *in silico* mRNA structural modeling revealed local distortion near L144P. However, mRNA decay analysis confirmed that transcript stability was largely maintained or even increased in these cells, indicating that changes in mRNA stability alone are unlikely to fully explain the reduced channel function 62. Instead, tRNA sequencing revealed a low abundance of the cognate tRNA for the synonymous 'TTA' Leu codon (CPM = 1698.2) among all Leu-tRNAs, suggesting that 'TTA' is not the preferred ('optimal') codon and may cause ribosome stalling, altered elongation rate during translation, or chaperone-assisted co-translational protein folding 63. In addition to mRNA/tRNA-dependent mechanisms, additional factors likely include increased degradation of misfolded Kir7.1, defective trafficking, and subtle conformational changes caused by bystander nucleotide substitutions. Collectively, these mechanisms may limit functional rescue despite effective genomic editing, underscoring the need to further optimize precision editing strategies.

The therapeutic relevance of precise correction is supported by the BRILLIANCE (EDIT-101) clinical trial (NCT03872479), which predicted that editing approximately 10% of foveal cones could restore near-normal vision in LCA10 patients 60. For correcting L144P and similarly complex loci, CBEs could be evolved or engineered to have a more precise editing window 47, 64 with fewer insertion-deletion mutations (indels), thereby increasing the “CTA” codon outcomes (which only 3% of BE4max edited cells showed as wild-type genotype) to at least 10%, a level we believe would produce beneficial clinical outcomes. Alternatively, other editing strategies can be considered.

Prime editing achieved ~20% efficiency (CCA>CTA) at the L144P locus without any synonymous or bystander nucleotide changes and restored Kir7.1 channel function in 28% of the evaluated cells, indicating a mixed population of cells with monoallelic and biallelic edits. Notably, ~18% of the edited reads contained single-nucleotide insertions, a byproduct that could be minimized in the future. Although there is scope to further improve prime editing efficiency by testing various ngRNAs in combination with multiple refined pegRNAs 54 and by adopting the PE4/PE5 system to reduce indel formation, our results demonstrate that prime editing can overcome the sequence constraints that limit CBE-based correction at the locus.

In summary, our study produced several key findings. We demonstrated that the L144P mutation severely impairs Kir7.1 channel activity in hiPSC-RPE cells. Developing an L144P hiPSC-RPE model using LNP-mediated delivery of an adenine base editor advances this research beyond a simple proof of concept toward a more practical application. We demonstrated that although CBEs can efficiently target the *KCNJ13* L144P locus, their therapeutic utility is limited by bystander editing and Kir7.1's functional sensitivity to synonymous variation. For mutation sites such as L144P, a "customized" selection of the optimal editing tool is necessary. This experience is broadly applicable to selecting therapeutic strategies for other similar loci. Prime editing enables precise correction without these limitations and therefore represents a more suitable strategy for treating L144P-associated LCA16. While intentionally introducing silent bystander edits may boost prime editing efficiency 52, our results suggest caution to prevent potentially harmful consequences of silent editing. These findings underscore the importance of functionally validating edited alleles in early-stage preclinical studies before advancing to late-stage translational endeavors. Our findings suggest that future therapeutic advances may require greater editing precision, codon-aware editing strategies, or refined delivery approaches to prevent vision loss in patients with LCA16.

### Limitations of the study

We also acknowledge several limitations. First, genome-editing efficiencies and functional outcomes measured *in vitro* may not fully reflect the *in vivo* retinal physiological and physical challenges. Therefore, it is crucial to evaluate this strategy in more clinically relevant systems, such as hiPSC-RPE or *in vivo* mouse models. Second, future studies should systematically assess the functional impact of indel-containing alleles, as their relative abundances, specifically the ratio of Leu-edited alleles to indel or unedited alleles, within individual CBE-edited cells may affect overall channel function. Third, RNA off-target analyses were not performed because Kir7.1 channel function was lost following CBE treatment. DNA off-target analyses were limited to predicted sites and may not detect rare or context-dependent off-target events, especially in primary retinal cells. Additionally, while our data suggest that synonymous codon usage and tRNA availability could contribute to impaired channel function, we did not directly manipulate codon usage or tRNA levels to establish causality. Finally, prime-editing efficiencies remain modest and require further optimization to reach therapeutically relevant correction levels *in vivo*. Overcoming these limitations in future research will be crucial for advancing genome-editing therapies for LCA16 toward clinical applications.

## Supplementary Material

Supplementary figures and tables.

## Data Availability

All relevant raw data will be freely available to any researcher wishing to use them for non-commercial purposes without breaching participant confidentiality.
