# Supplementary material for: Synonymous editing alters ion channel function, favoring prime editing for retinal disease correction
Source: Int J Biol Sci. 2026 Apr 23;22(9):4670–90. doi: 10.7150/ijbs.132743 (PMC13182245; doi:10.7150/ijbs.132743)
Supplement: Supplementary file 1 — Supplementary figures and tables. [file ijbsv22p4670s1.pdf]

Synonymous editing alters ion channel function, favoring prime editing for retinal disease correction

Meha Kabra[1, 2#], Mariya Moosajee[3, 4, 5], Ana Navarrete[6], Gregory A. Newby[7, 8,9], Piper Rawding[2,10,11], Alex Xie[2,10,11], Hadas Mechoulam[6], Antonio Rivera[6,] Alan Hung[1], Smriti Tiwari[1], Adam J. Waxman[1], Kaivalya Molugu[12], Krishanu Saha[1,2, 10,12,13], Shaoqin Gong[2,10,11], David R. Liu [7,8,9], Bikash R. Pattnaik [1, 2, 11#]

1. Department of Pediatrics, University of Wisconsin-Madison, Madison, WI 53792, United States.
2. McPherson Eye Research Institute, University of Wisconsin-Madison, Madison, WI 53705, United States.
3. Development, Ageing, and Disease, University College London (UCL) Institute of Ophthalmology, London, EC1V 9EL, United Kingdom.
4. Department of Genetics, Moorfields Eye Hospital Health Services (NHS) Foundation Trust, London EC1V 2PD, United Kingdom.
5. Ocular Genomics and Therapeutics Laboratory, The Francis Crick Institute, London, NW1 1AT, United Kingdom.
6. Department of Ophthalmology, Hadassah Medical Center, Jerusalem, Israel
7. Merkin Institute of Transformative Technologies in Healthcare, Broad Institute of Harvard and MIT, Cambridge, MA 02142, United States.
8. Howard Hughes Medical Institute, Harvard University, Cambridge, MA 02138, United States.
9. Department of Chemistry and Chemical Biology, Harvard University, Cambridge, MA 02138, United States.
10. Wisconsin Institute of Discovery, University of Wisconsin-Madison, Madison, WI 53715, United States.
11. Department of Ophthalmology and Visual Sciences, University of Wisconsin-Madison, Madison, WI 53705, United States.
12. Department of Biomedical Engineering, Wisconsin Institute of Discovery, University of Wisconsin-Madison, Madison, WI 53706, United States.
13. Center for Human Genomics and Precision Medicine, University of Wisconsin-Madison, Madison, WI 53705, United States.

#Corresponding authors
Meha Kabra, Email: **mkabra2@wisc.edu**
Bikash R. Pattnaik, Email: **pattnaik@wisc.edu**

Supplementary

Supplementary Table 1: Primers to amplify the on-target and off-target sites

	Primer name		Sequence (5'-3')	GC %	Amplicon size
**On-Target**	L144P hiPSC-RPE-creation	FP	CCCTGGAGACACAACTCACA	55	200
		RP	ACTATACCCTTTCCAAACACCTG	43	
**On-Target**	L144P	FP	CCCTGGAGACACAACTCACA	55	147
		RP	GGGCAATCTTCGCCACAAAA	50	
**OT1**	Intergenic-AC093639.1-MIR548AE1	FP	CCCTGGAAGAAGTAATACAT	40	133
		RP	ACCTATTTAGCTTGGATCTC	40	
**OT2**	Intron-ZC3H3	FP	GGACACAGGGACGCAGAT	61	231
		RP	AGACAATGGGGAGAAAGCCT	50	
**OT3**	Intron-PDZD4	FP	AAGGCCCTGGCTACTCACAG	60	146
		RP	TGAGTCAGTACAGCGCCACC	60	
**OT4**	Intergenic-IL22-MDM1	FP	TGCAGCCTGAGGATTACAGA	50	160
		RP	TGTACCCCCATTTCGACACC	55	
**OT5**	Intergenic-RP5-823G15.5-Y_RNA	FP	CCACTTGACATGTAGACCTGA	47	147
		RP	AGGTCCCTAAAGTTTGCACAT	47	
**OT6**	Intergenic-RHBDD2-POR	FP	GATTCTGTGCCAAGCCGGAG	60	125
		RP	AAGGGCGGGCATCACCTATT	55	
**OT7**	Intergenic-AC005775.2-TPGS1	FP	ACTTCCTCTCTGGGACCCTT	55	152
		RP	CCTGGGTGGGCTGCTTAAC	63	
**OT8**	Intron-DCDC2	FP	CTCAATCCTCATGCTAGCCCT	52	143
		RP	CCCCTGACTGGCTACAGGAT	60	
**OT9**	*GRCh38-80320077	FP	CCTCTGTCTGTTGCTGACAT	50	174
		RP	ACCGGAGAAGAAGCTGTGAT	50	
**OT10**	*GRCh38-6078211	FP	ACTCCATAGGAGCAGGTTTCTG	50	172
		RP	TCCTTAGGGGTAGAGGCCAT	55	
**OT11**	GRCh38-45165776	FP	AGCTTCCACACCCTCTGTTT	50	149
		RP	AGTAAAGGAGCTGGCCCAAG	55	
**OT12**	GRCh38-96006488	FP	ACACCCAAATTGACCAGCAG	50	144
		RP	GGCTAATCAAGGCTCTGGAA	50	

The primers were designed using the NCBI Primer BLAST tool and ordered from IDT with an Illumina NGS platform adaptor sequence. OT = off-targets. Adaptor sequence for FP: 5'-ACACTCTTTCCCTACACGACGCTCTTCCGATCT-3'. Adaptor sequence for RP: 5’-GTGACTGGAGTTCAGACGTGTGCTCTTCCGATCT-3'. The adaptors were interchanged for the primers marked with *.

Supplementary Table 2: Primers for in-fusion cloning of *KCNJ13* in FLP-In™ expression vector

Primer name	Sequence (5'-3')	GC %
FRT_FP	TCACTATAGGGAGACCCAAGCTGGCTAGCGTTTAAACTTAatggtgagcaagggcgagga	50
FRT_RP	AGTCGAGGCTGATCAGCGGGTTTAAACGGGCCCTCTAGACttattctgtcagtcctgttt	50

FP: Forward primer, RP: Reverse primer. Primers were designed using the Gibson Assembly primer design tool available at https://tools.sgidna.com/gibson-assembly-primers.html and ordered from IDT (https://www.idtdna.com). The homology sequence is in uppercase, and the annealing sequence is in lowercase.

Supplementary Table 3: Primers for Sanger sequencing

Primer name	Sequence (5'-3')	GC%	Tm [predicted]
GFP FP	CAAGTCCGGACTCAGATCTCGAGCTC	57.1	72.8
Kir7.1 RP	TTATTCTGTCAGTCCTGTTT	72.7	90.9

The primers were designed using the NCBI Primer BLAST tool (https://www.ncbi.nlm.nih.gov/tools/primer-blast/).

Supplementary Table 4: Potential off-target sites for L144P sgRNA location screened by deep sequencing

Off-target	Gene/Region/Location	Chromosome	Strand	Mismatches	Bulge size/ Bulge type
1	Intergenic: AC093639.1-MIR548AE1	2	-	2	-
2	Intron: ZC3H3	8	+	2	-
3	Intron: PDZD4	X	+	3	-
4	Intergenic: IL22-MDM1	12	-	3	-
5	Intergenic: RP5-823G15.5-Y_RNA	20	-	4	-
6	Intergenic: RHBDD2-POR	7	-	4	-
7	Intergenic: AC005775.2-TPGS1	19	-	3	-
8	Intron: DCDC2	6	+	3	-
9	GRCh38: 80320077	8	+	2	1/ DNA
10	GRCh38: 6078211	17	+	1	1/ RNA
11	GRCh38: 45165776	15	-	2	1/ DNA
12	GRCh38: 96006488	8	+	2	1/ RNA

Supplementary Table 5: Nicking guide RNA sequences used for prime editing

Name	Sequence 5’-3’	PAM
Nicking guide RNA 1	TATGGTACCATGTTCCCCAG	TGG
Nicking guide RNA 2	ACACAACTCACAATTGGTTA	TGG

Supplementary Table 6: DNASTAR (protean-3D) predicted differences in the secondary structure (dihedral angles) of Kir7.1

Genotype	φ	ψ	ω
WT	-67.1°	-34.6°	170.7°
L144P	-62.8°	-36.5°	173.8°

Supplementary Table 7: SOPMA predicted differences in the secondary structure of Kir7.1

Genotype	alpha helix	beta-turn	extended strand	random coil
WT	30.56%	4.72%	23.33%	41.39%
L144P	29.44%	5.28%	22.50%	42.76%

Supplementary Figures

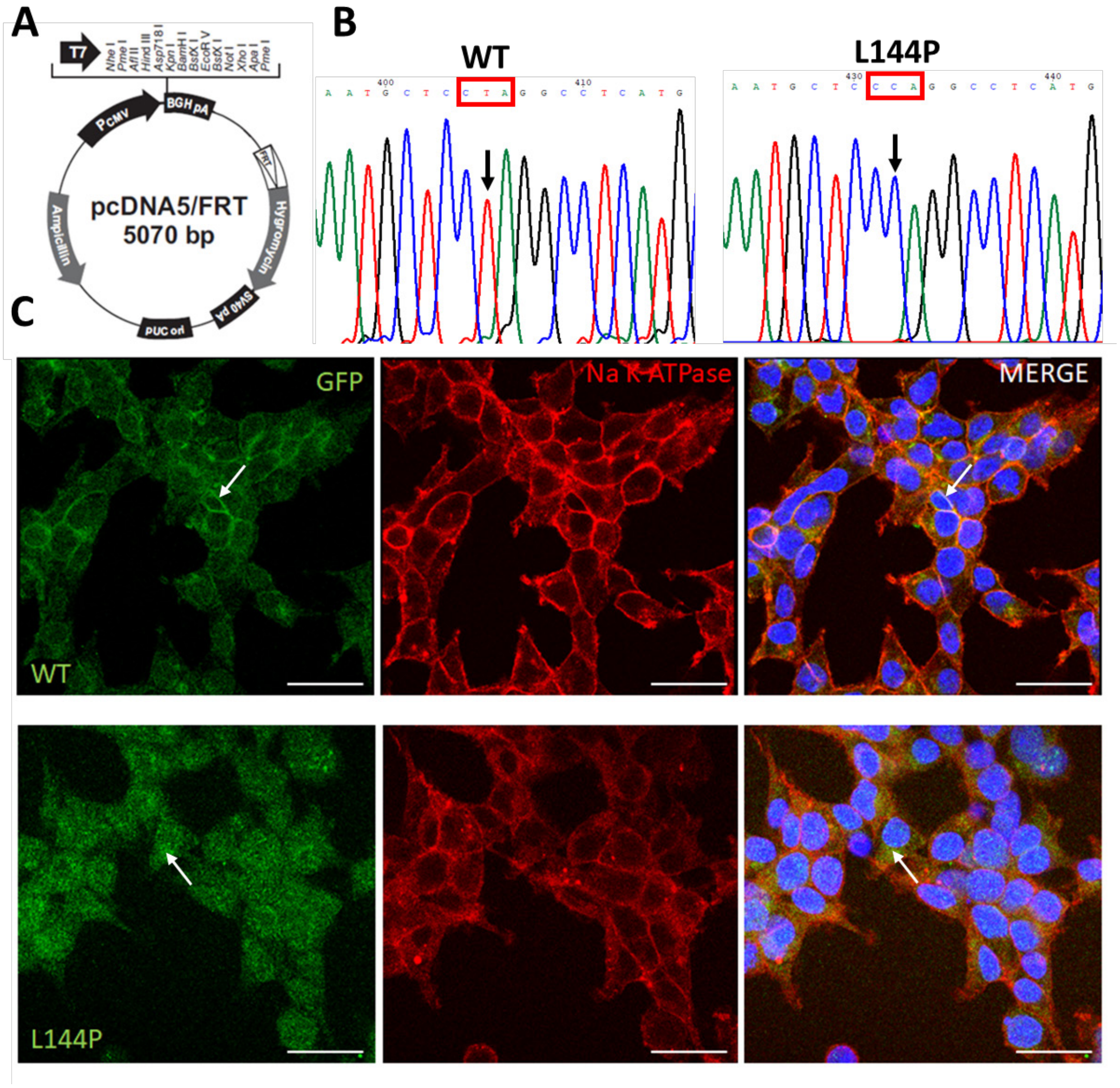

Supplementary Figure 1: Characterization of HEK293 FRT WT and L144P stable cells. [A] FLP-In™ expression vector map used for in-fusion cloning to express WT and L144P Kir7.1. [B] WT and L144P mRNA sequences from the respective HEK293 FRT stable cells. [C] Native and L144P Kir7.1 protein expression in stable cells assessed by immunocytochemistry. Scale: 25 µm.

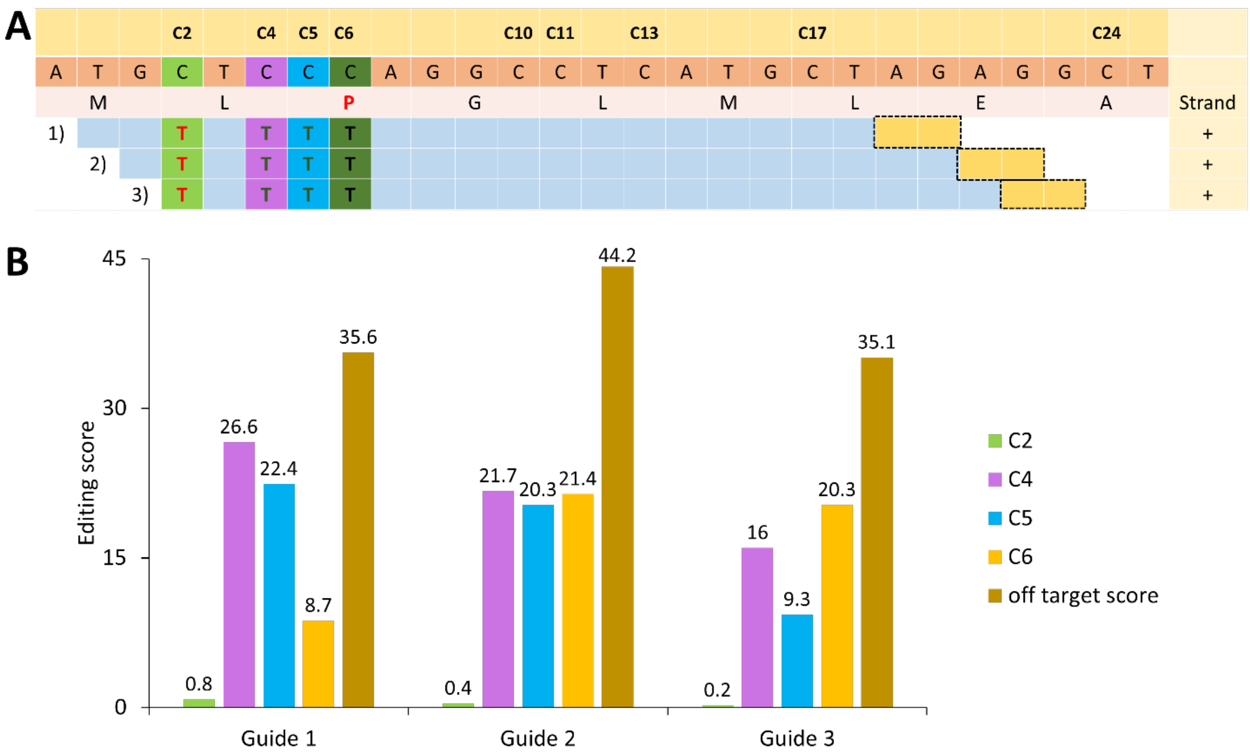

Supplementary Figure 2: The gRNA design and selection. [A] Three gRNAs with an NG PAM (black-dashed rectangle) located in the KCNJ13 gene sequence. [B] On-target scores at different neighboring Cs and off-target scores for three guides designed using the Benchling software.

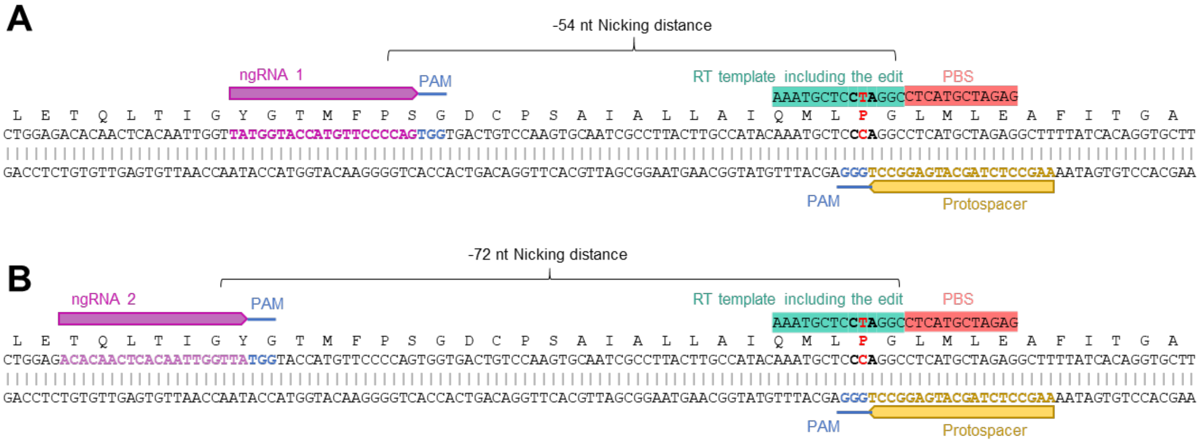

Supplementary Figure 3: Design and location of pegRNA and ngRNAs. [A] Design of PE3 editing using ngRNA 1 to correct the L144P mutation in the *KCNJ13* gene in HEK293 stable cells. [B] Design of PE3 editing using ngRNA 2 to correct the L144P mutation in the KCNJ13 gene in HEK293 stable cells.

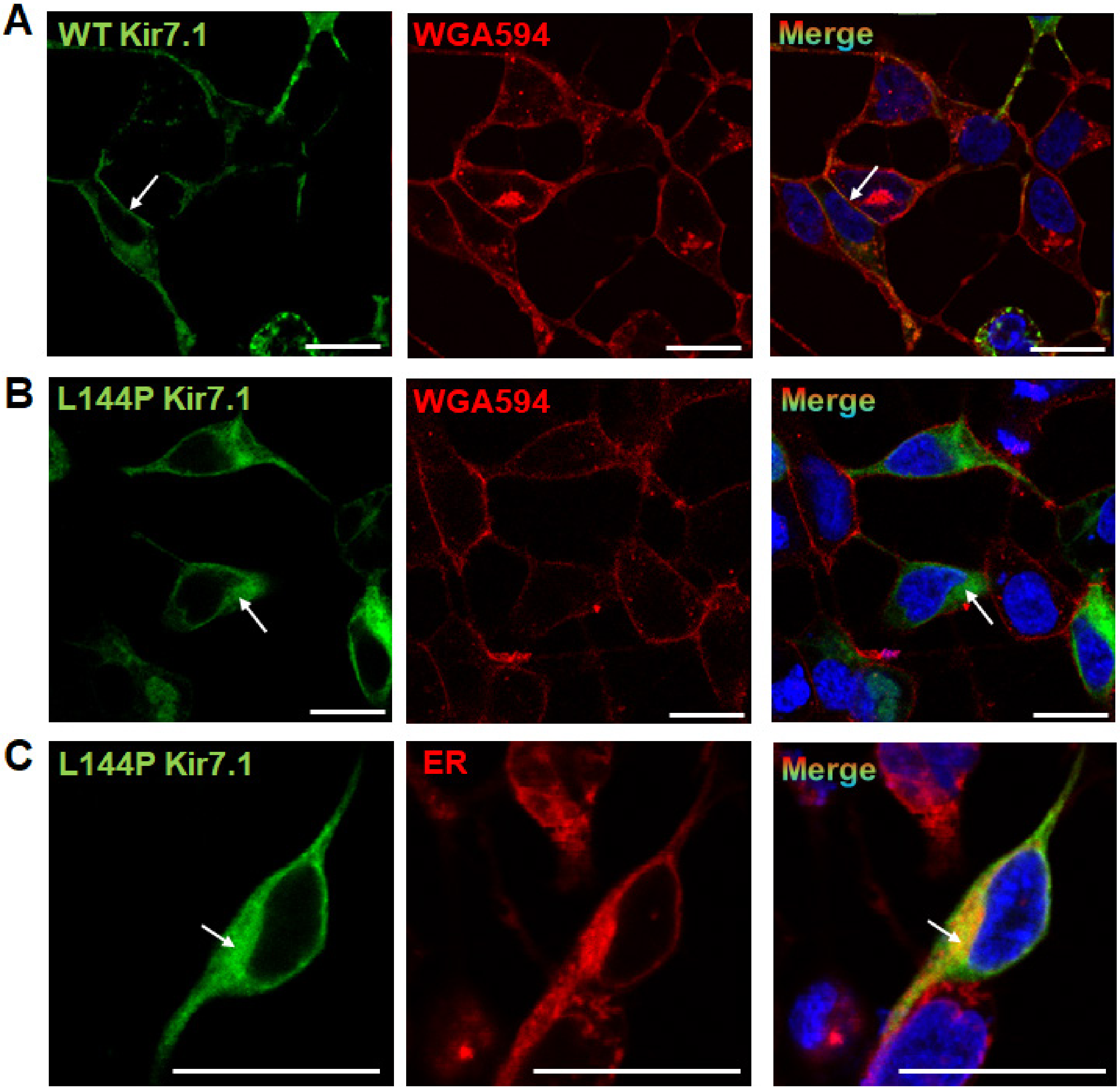

Supplementary Figure 4: Localization of the mutant Kir7.1 protein in HEK293 cells. The cells were transfected with GFP-WT-Kir7.1 or GFP-L144P-Kir7.1 plasmids. [A] Native Kir7.1 (green) expression at the membrane (red). [B] Mutant L144P-Kir7.1 (green) expression in the cytoplasm and other organelles. [C] Localization of a significant proportion of L144P-Kir7.1 in the Endoplasmic Reticulum (red). White arrows indicate colocalization of Kir7.1 with the membrane, cytoplasm, or ER. Scale: 25 μm.

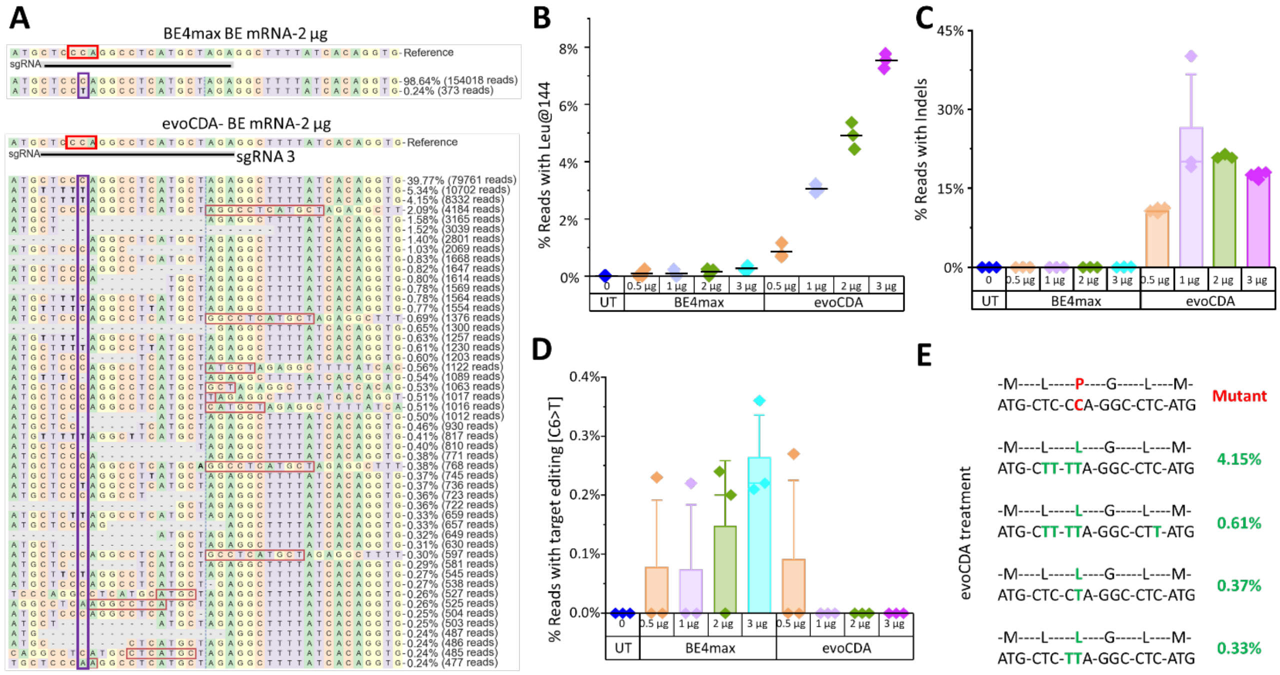

Supplementary Figure 5: Evaluation of sgRNA-3 activity with BE4max and evoCDA mRNA at the L144P locus. [A] Sequencing reads from sgRNA3 and BE4max- or evoCDA-treated cells showing the nucleotide distribution around the cleavage site (a dashed vertical line) for sgRNA. The sgRNA location is underlined, the target location C6 is marked with a purple box, and the mutant codon is marked with a red rectangle. A dashed line '-' indicates base deletions, a maroon rectangle indicates insertions, and substitutions are shown in bold. BE4max mRNA-treated cells showed only mutant and on-target C6>T edited reads. A variety of reads were generated from evoCDA mRNA-treated cells at different frequencies. [B] % of sequencing reads with on-target and/or synonymous bystander edits resulting in an aa Leu at position 144. Data were generated in three independent experiments by treating the cells with different doses of BE4max or evoCDA mRNA. [C] % of sequencing reads with indels from cells treated with different doses (0.5, 1, 2, 3 µg) of BE4max and evoCDA mRNA. [D] % of sequencing reads with on-target C6>T editing generated from cells treated with different doses (0.5, 1, 2, 3 µg) of BE4max and evoCDA mRNA. [E] Amino acid conversion at the respective location for reads generated from evoCDA treatment resulted in Leu at position 144. Figures presenting pooled data are represented as mean ± SEM (n=3).

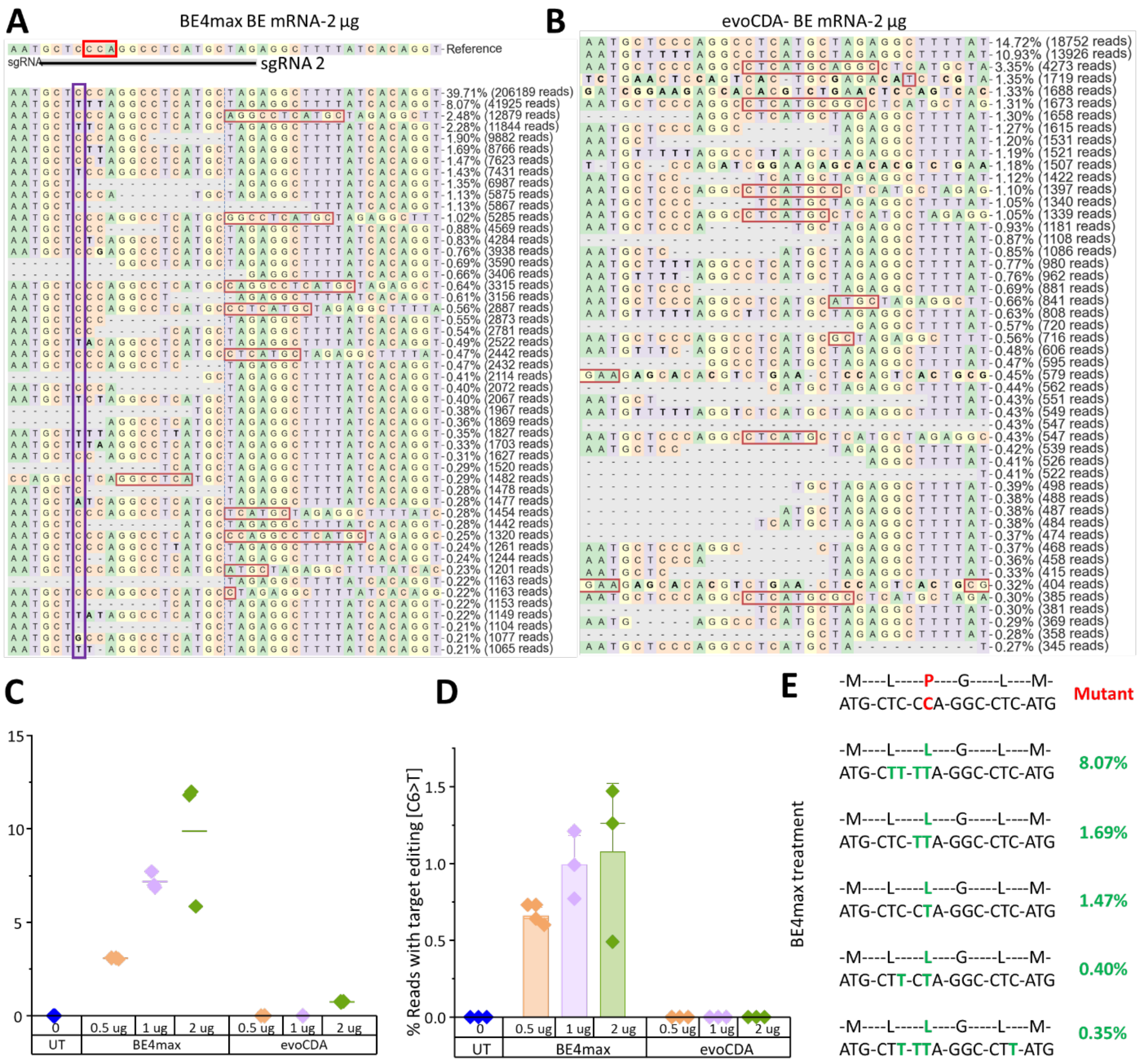

Supplementary Figure 6: Evaluation of different doses of CBE mRNA activity with sgRNA-2 at the L144P locus. [A] Sequencing reads from sgRNA-2 and BE4max-treated cells showing the nucleotide distribution around the cleavage site (a dashed vertical line) for sgRNA. The sgRNA location is underlined, the target C6 is marked with a purple box, and the mutant codon is marked with a red rectangle. A dashed line '-' indicates base deletions, a maroon rectangle indicates insertions, and substitutions are shown in bold. BE4max mRNA-treated cells showed a variety of reads with Leu at position 144. [B] A variety of sequencing reads from sgRNA-2 and evoCDA-treated cells showing the nucleotide distribution around the cleavage site. [C] % of sequencing reads with on-target and/or synonymous bystander edits resulting in an aa Leu at position 144. Data were generated in three independent experiments by treating the cells with different doses (0.5, 1, 2 µg) of BE4max or evoCDA mRNA. [D] % of sequencing reads with on-target C6>T editing generated from cells treated with different doses (0.5, 1, 2 µg) of BE4max and evoCDA mRNA. [E] Amino acid conversion at the respective location for reads generated from BE4max mRNA-treated cells resulted in Leu at position 144. Figures presenting pooled data are shown as the mean ± SEM (n = 3).

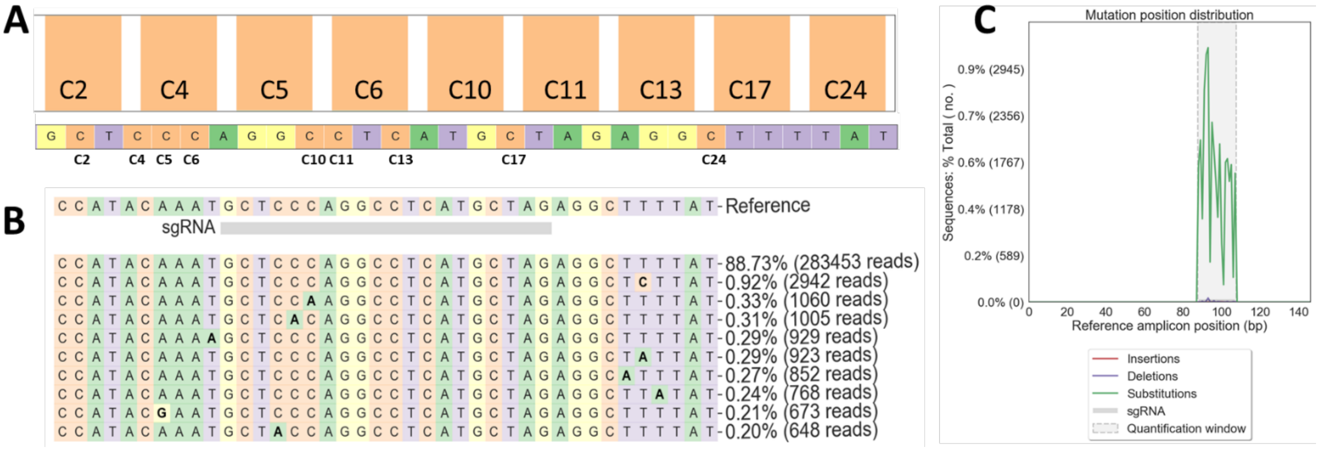

Supplementary Figure 7: Sequencing readouts from untreated L144P stable cells used as a reference. [A] Nucleotide distribution around the sgRNA location as observed in sequencing reads. [B] Percentage of sequencing reads in the untreated sample. [C] Percentage distribution of substitutions and deletions at the sgRNA location.

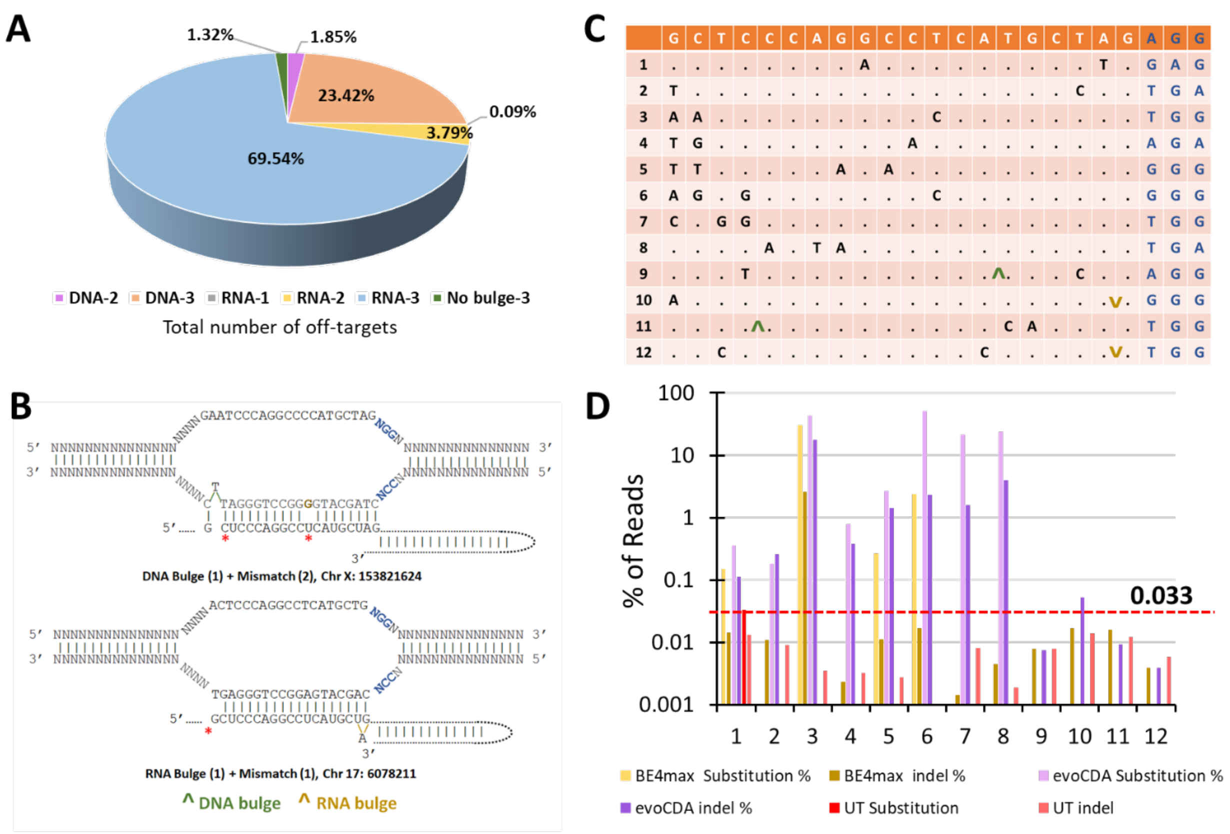

Supplementary Figure 8: Off-target analysis of the L144P sgRNA in CRISPR base-edited cells. [A] Total number of off-targets, each with 1-3 mismatches, with or without a single RNA/DNA bulge, as observed in in silico analysis. (Figure 6_Source Data 1 contains the complete list of off-targets.) A large fraction of OT sites had 3 mismatches and a single RNA bulge, followed by 3 mismatches and a single DNA bulge. ['DNA-2' in the pie chart represents the single DNA bulge with 2 mismatches, 'RNA-3' represents the single RNA bulge with 3 mismatches, and so on.] [B] Representation of DNA and RNA bulges with 1 or 2 mismatches relative to the L144P sgRNA-2. [C] The 12 potential off-target sites with mismatches and DNA/RNA bulges and PAM sites were screened by deep sequencing. [D] % substitution and indel frequency of BE4max and evoCDA CBEs at 12 off-target sites (Figure 6_Source Data 2 contains the NGS files in fastaq.gz format). L144P cells sham-nucleofected were used as a reference. A threshold (red dashed line) was set at 0.033 based on base-level substitutions and indels in reference cells.

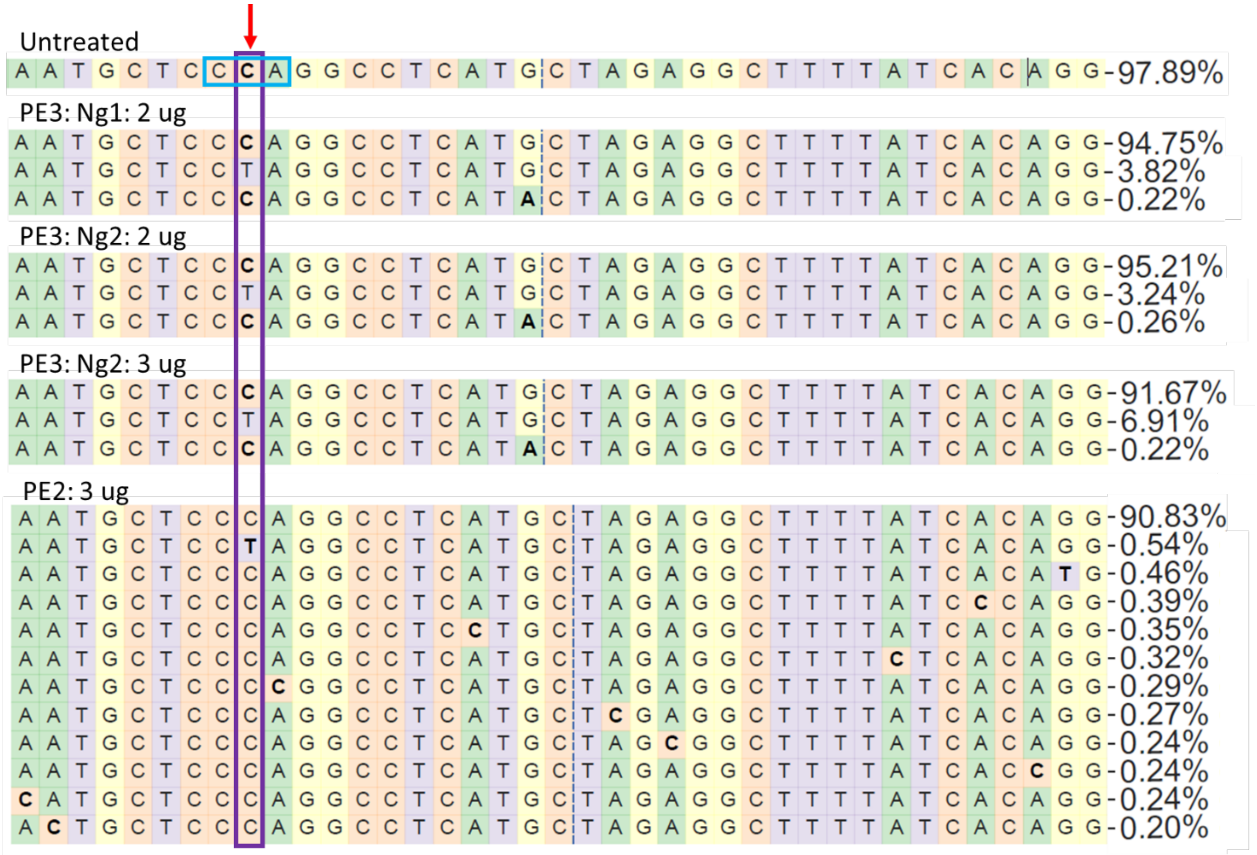

Supplementary Figure 9: Prime editing efficiency using the PE2 and PE3 strategies. On-target editing efficiency at the L144P locus using two nicking guide RNAs and two doses of pegRNA. The top few reads show the nucleotide distribution around the on-target ‘C’ base. A red downward arrow highlights the target ‘C’ base, and the mutant codon is shown in a blue box. Substitutions are shown in bold. Each treatment’s read results show the desired C>T conversion and its corresponding frequency.
